# Genome-scale insights into the metabolic landscape and evolutionary development of *Bifidobacterium bifidum*

**DOI:** 10.20517/mrr.2026.19

**Published:** 2026-08-12

**Authors:** Emanuele Selleri, Giulia Longhi, Chiara Tarracchini, Erica de Carolis, Silvia Petraro, Leonardo Mancabelli, Christian Milani, Francesca Turroni, Marco Ventura, Gabriele Andrea Lugli

**Affiliations:** ^1^Laboratory of Probiogenomics, Department of Chemistry, Life Sciences, and Environmental Sustainability, University of Parma, Parma 43124, Italy.; ^2^Department of Medicine and Surgery, University of Parma, Parma 43124, Italy.; ^3^Microbiome Research Hub, University of Parma, Parma 43124, Italy.

**Keywords:** Bifidobacteria, genomics, metagenomics, microbiome

## Abstract

**Background:** *Bifidobacterium bifidum* (*B. bifidum*) is an infant gut symbiont specialized in degrading host-derived glycans. Despite its relevance in early life, the species’ genomic diversity has not yet been comprehensively surveyed, and current reference collections capture only a fraction of the global *B. bifidum* pangenome.

**Methods: **In this study, we reconstructed the first comprehensive pangenome of *B. bifidum* using 1,351 high-quality genomes, including metagenome-assembled genomes. This dataset was used for *in silico* comparative genomics analyses to identify species-specific genetic and functional features. *In vitro* transcriptomics analyses were further performed to validate and functionally characterize selected species-specific traits.

**Results: **Comparative genomic analysis with other human-associated bifidobacteria species identified 667 *B. bifidum*-specific clusters of orthologous genes mostly involved in carbohydrate utilization, osmotic regulation, and host interaction. Notably, *B. bifidum* displays the most extensive enzymatic repertoire for host-glycan degradation, dedicating 43% of its conserved glycoside hydrolases to these substrates. We identified significant gain-of-function events, including two unique phosphotransferase systems (PTS) for disaccharide uptake. Transcriptomic profiling corroborated the functional relevance of these PTS clusters, which were significantly up-regulated during growth on human milk oligosaccharides, mucin, and N-acetylglucosamine. While the species exhibits high genomic stability, a localized divergence (average nucleotide identitiy, ANI < 98.5%) was identified in rural, non-Westernized populations, reflecting niche-specific adaptations.

**Conclusion:** The identified genomic framework highlighted a distinct evolutionary path of *B. bifidum*, placing this taxon as a metabolic cornerstone in the neonatal gut via extensive metabolic specialization toward glycan hosts.

## INTRODUCTION

*Bifidobacterium bifidum* (*B. bifidum*) is a Gram-positive, high Guanine-Cytosine (GC)-content, anaerobic bacterial species belonging to the phylum Actinomycetota^[1]^. This species is a relevant member of the human gut microbiota from early life, where it plays a pivotal role in maintaining host homeostasis by modulating immune responses, inhibiting pathogenic organisms, and supporting the structural integrity of the intestinal barrier^[2-5]^. In contrast to widely distributed species such as *Bifidobacterium adolescentis* and *Bifidobacterium longum*, which are the most abundant bifidobacterial taxa within the gut microbiota of adult hosts, *B. bifidum* is characteristic of the bifidobacterial communities residing in the infant gut and contributes to early microbial colonization, alongside *Bifidobacterium breve*^[6-10]^. *B. bifidum* can be considered a human-associated bifidobacterial species (HBS), alongside *B. adolescentis* and *B. longum*, but also with *Bifidobacterium catenulatum*, *Bifidobacterium dentium*, and *Bifidobacterium pseudocatenulatum*^[9,10]^.

*B. bifidum* exhibits highly specialized metabolic pathways adapted to the unique nutritional and ecological conditions of the neonatal gut environment. In contrast to most HBS, which primarily metabolize plant-derived glycans, *B. bifidum* degrades host-derived complex carbohydrates, including mucin and intestinal epithelial glycans^[11,12]^. This enzymatic capacity enables the de-sialylation of host structures, such as the transmembrane mucin MUC13, thereby enhancing intestinal barrier function and conferring a stable ecological advantage^[12]^. By metabolizing human milk oligosaccharides (HMOs), *B. bifidum* functions as a supporting metabolic hub, enhancing the growth of other key commensals, such as *Faecalibacterium prausnitzii*^[13,14]^. Moreover, *B. bifidum* harbors distinct mechanisms for exploiting nitrogen and lipid sources, including peptides and whey protein-derived phospholipids, which likely confer a competitive advantage in the nutrient-rich neonatal environment^[15,16]^. 

These metabolic specializations are associated with genetic trade-offs, including amino acid auxotrophy, which reflect adaptation to a nutrient-rich host environment^[17]^. The ecological success of *B. bifidum* may largely be attributed to its ability to balance structural adaptations with defensive mechanisms. In particular, the production of extracellular polysaccharides, such as ß-glucan/galactan^[18,19]^, and specialized surface structures like sortase-dependent pili and teichoic acids^[20-23]^, play a pivotal role in biofilm architecture and mediating host interactions, thereby contributing to the modulation of host immune responses. In addition, the presence of Clustered Regularly Interspaced Short Palindromic Repeats (CRISPR)-Cas systems and multiple restriction-modification systems confers protection against mobile genetic elements, contributing to genome stability and shaping evolutionary trajectories^[20,24]^.

At a phylogenetic level, *B. bifidum *differs from other HBS, which cluster into two specific subgroups, i.e., the* B. adolescentis group *and the* B. longum* group, whereas *B. bifidum* forms a separate phylogenetic group of its own^[25]^. The phylogenetically related *B. bifidum* species (PBS), collectively forming the *B. bifidum* group, include* B. aerophilum, B. amazonense, B. biavatii, B. goeldii, B. hapali, B. jacchi, B. leontopitheci, B. miconis, B. ramosum, B. samirii, B. scardovii,* and* B. simiiventris*, are predominantly inhabitants of the non-human primates' gut^[25-28]^. Remarkably, unlike other HBS, *B. bifidum* has followed a distinct evolutionary trajectory, reflecting a specialized adaptation to the human gut.

The genetic determinants of *B. bifidum* remain surprisingly under-characterized, largely because previous pangenome efforts have been restricted to isolated, culturable strains. Comparative genomic studies involving *B. bifidum* have progressively shifted from characterizing its species-level genomic structure to resolving how niche, host age, and geography shape its diversity. An early phylogenomic survey of 95 *B. bifidum* strains confirmed that the species forms a genetically coherent group, clearly distinguishable from other *Bifidobacterium* taxa and sharing a large proportion of orthologous gene families^[29]^. Within a subset of 15 strains representing distinct human body niches, such as gut, vagina, and breast milk, the carbohydrate-active enzyme repertoire varied consistently with isolation site, pointing to niche-specific metabolic adaptation. A larger survey of 140 strains isolated from Chinese adult and infant feces later confirmed an open pangenome and traced most of the observed intraspecific diversity to carbohydrate metabolism and defense-related functions, including glycoside hydrolase content, bacteriocin operons, antibiotic-resistance genes, and CRISPR-Cas systems, indicating the type III-A CRISPR-Cas locus as characteristic of this taxon, with phylogenetic clustering tracking both geographic origin and host age^[30]^. The contribution of host age was addressed more directly in a comparison of six strains from distinct ages, in which strains from children and young hosts showed enhanced degradation of 6’-sialyllactose, an HMO component, relative to strains from adults^[31]^. More recently, a comparison of *B. bifidum* genomes isolated from Chinese, Russian, and American populations reported a core-genome phylogeny structured by geographic origin, with variation in carbohydrate-active enzyme copy number consistent with a dietary contribution to this divergence^[32]^. Collectively, these studies show that isolation niche, host age, and geography each leave a discernible signature on *B. bifidum* genomic and functional diversity. However, all datasets examined so far have been restricted to isolated, culturable strains drawn from a narrow set of cohorts (China, Russia, North America), leaving unresolved the extent to which the species’ diversity remains uncharacterized, particularly in non-Westernized rural populations. As recently observed with the *B. adolescentis* and *B. longum* pangenome reconstruction, the inclusion of metagenome-assembled genomes (MAGs) provides a far more complete genetic landscape^[33,34]^. Here, we collected 1,351 high-quality genomes from isolated cultures and metagenomic samples to highlight the unique functional traits of* B. bifidum* relative to other HBS and to emphasize its evolutionary trajectory among PBS. We further assessed the functional relevance of selected traits through transcriptomic profiling of *B. bifidum* PRL2010 grown on host-derived substrates, including mucin, HMOs, and N-acetylglucosamine (GlcNAc). These experiments revealed how *B. bifidum* modulates gene expression of its unique PTS systems to thrive in its specialized ecological niche.

## MATERIALS AND METHODS

### Bacterial isolation and growth conditions

*B. bifidum* was isolated from stool samples and preserved in the culture collection of the Probiogenomics Laboratory (University of Parma, Italy). Stool samples were diluted in physiological solution and plated on Man-Rogosa-Sharpe (MRS) agar (Scharlau Chemie, Spain) containing 0.05% (w/v) L-cysteine hydrochloride and mupirocin (50 mg/L) and incubated in a specialized chamber (Concept 400; Ruskin) under anaerobic conditions (2.99% H_2_, 17.01% CO_2_, and 80% N_2_) at 37 °C for 48 h. For subsequent assays, *B. bifidum* cells were grown under the same anaerobic conditions in MRS broth. The genome of six *B. bifidum* strains were decoded in the framework of this study and deposited in GenBank as strains 81B, 98B, 184B, 352B, 361B, and 2350B, under NCBI accession numbers GCF_055790765.1, GCF_055790725.1, GCF_055790805.1, GCF_055790825.1, GCF_055790785.1, and GCF_055790745.1, respectively.

### Genome assembly

Genome assemblies were generated from sequencing data of *B. bifidum* isolates in the Laboratory of Probiogenomics collection and from public shotgun metagenomic data of the human gut microbiota. Locally isolated reads were assembled with MEGAnnotator2, while new metagenomes were obtained from public repositories using the METAnnotatorX2 pipeline. SPAdes version 4.3.0 was used in both pipelines, with either “--isolate” or “--meta” invoked, depending on the sample. For both software tools, the default parameters were utilized^[35-37]^.

### Genomic datasets

The *B. bifidum *dataset comprised all publicly available *B. bifidum *genomes retrieved from NCBI, together with an additional 1,106 MAGs reconstructed from 86 projects. Finally, six new genome assemblies derived from bacterial isolates from the Probiogenomics Laboratory collection were added [Supplementary Table 1]. The quality of each genome was evaluated using CheckM2, based on completeness and contamination levels^[38]^. Genomes with completeness below 95% and/or contamination above 5% were discarded. Dereplication of the dataset was performed using dRep, with a 99.99% threshold to remove genomic redundancy^[39]^. The final *B. bifidum* dataset includes 1,351 unique genomes [Supplementary Table 1]. We also downloaded all high-confidence genome sequences of HBS and PBS from NCBI^[40]^ [Supplementary Table 2].

### Horizontal gene transfer events of the *B. bifidum* species

The significant divergence in codon usage bias (CUB) and GC content was analyzed to identify highly confident horizontal transfer gene (hcHTG) between clusters of orthologous genes (COGs) from the RefSeq NCBI genomes of *B. bifidum*^[41]^. The value of CUB was influenced by: Relative Synonymous Codon Usage (RSCU), which is the codon usage compared to the theoretical maximum, Effective Number of Codons (ENC), indicating the specificity required by the gene in the codon usage, the Codon Adaptation Index (CAI), which considers the codon usage compared to highly expressed reference genes, and GC content (expressed as a within-genome z-score). Genes in which all four parameters deviated from the 90% of the genome gene values (above the 90th percentile for RSCU, ENC and GC content and below the 10th percentile for CAI, respectively) were therefore classified as high-confidence horizontally transferred genes (hcHTGs). Percentile thresholds were computed independently for each genome. Across all analyzed *B. bifidum* genomes, the average threshold values were 0.49 ± 0.01 for the CAI 10th percentile, 34.26 ± 0.39 for the 90th percentile of ENC, 1.28 ± 0.05 for the GC content z-score 90th percentile, and 0.987 ± 0.00 for the RSCU 90th percentile.

Each COG formed by hcHTG was classified as a putative acquired COG. To identify putative COG donors, a BLASTP search was performed against the NCBI amino acid database, excluding matches to *B. bifidum* and *Bifidobacterium* sp. proteins. To compare the number of hcHTG events across the groups (*B. bifidum*, HBS, and PBS), a Kruskal-Wallis test, followed by pairwise Mann-Whitney *U* tests with Benjamini-Hochberg False Discovery Rate (FDR) correction, was performed. Furthermore, the internal distribution variability and homoscedasticity within each group were assessed and compared using the Fligner-Killeen test, also with FDR correction.

### CRISPR-Cas system and phage-targeting spacer analysis

CRISPR arrays and Cas gene clusters were predicted in each of the 1,351 *B. bifidum* genomes individually using CRISPRCasFinder v4.3.2^[42]^, run locally in “General” detection mode with the associated Cas-gene detection module. Each predicted CRISPR array was assigned a CRISPRCasFinder evidence level from 1 to 4, reflecting the confidence of the repeat-spacer architecture. All spacer sequences extracted from the predicted arrays were pooled and compared using the blastn-short task against the INPHARED phage genome database^[42]^. Matches were retained as significant when the percentage identity was ≥ 90%, the query coverage was ≥90%, and the e-value was ≤ 1e^-5^. For each validated hit, we collected the phage genome taxonomy, the corresponding CRISPR array, the CRISPRCasFinder evidence level of the source spacer, and the identity of the matched phage genome.

### Metabolic analyses

KEGG (Kyoto Encyclopedia of Genes and Genomes) codes for each COG^[43]^ were predicted using eggnog-mapper v2^[44]^. These annotations were used as input to the KEMET software^[45]^, which enables evaluation of the completeness of metabolic pathways for each genome. For both software, the default settings were used. Furthermore, the dbCAN3 software^[46]^ was used to assess the ability to utilize different carbon sources by identifying enzyme classes from the CAZy database^[47]^ involved in carbohydrate metabolism.

### Pangenome analyses

Gene annotations were performed by PROKKA^[48]^ with default settings. Pangenome studies were performed using the software Roary^[49]^, allowing the identification of the bifidobacterial core genome (genes present in 99% to 100% of the genomes in our dataset), soft-core genome (genes present in 95% to 99% of all genomes in the dataset), and accessory genome (genes present in less than 95% of all genomes analyzed). Genes’ distribution within Roary’s COGs was based on an identity threshold that varied by dataset (95% for single-species datasets and 80% for multispecies datasets).

The pangenome state was calculated using a custom Python 3 script that processed Roary’s output. The average pangenome progression of the dataset was computed from 1,000 possible permutations. For gene products not clearly annotated by Prokka, such as hypothetical proteins, a protein domain detection was performed with InterProScan using the Pfam 37.2 database^[50,51]^.

### Operon prediction in the genomes of *B. bifidum*

To identify potential operons comprising genes unique to *B. bifidum*, the PRL2010 strain upstream regions (nucleotides from position -200 to +70) of the unique *B. bifidum* COGs were analyzed. These regions were searched for RNA polymerase binding sites (RPBs) and transcription factor binding sites (TFBs), and their energetic and structural properties (ESp) were evaluated.

A reference database of RPB sequences was assembled from RNA-seq data reported in previous studies conducted on different strains of the genus *Bifidobacterium*^[52-56]^. For each reference sequence, all possible degenerate variants were generated by systematically replacing one or more nucleotide positions with an N wildcard, producing an exhaustive set of full-length and degenerate query patterns. These patterns were used to scan the upstream regions of all PROKKA-annotated genes for sequence matches. For the purposes of the present study, only exact matches (i.e., matches with zero substitutions) were retained as positive RPB calls. As a qualitative validation step, the matching procedure was applied to the well-characterized *B. breve* strain UCC2003^[52]^, confirming that the pipeline correctly identified the expected RPB sequences in the upstream regions of this strain, consistent with previously reported transcriptional data.

A custom database of TFBs was constructed using all available sequences deposited in BacRegDB^[57]^, and then used as a reference in BLASTN analyses of the upstream region sequences. The average ESp value for each dinucleotide was collected from previous studies and public online databases^[58-61]^. These values were used to calculate a continuous ESp score from 0 to 10 for each gene’s upstream region, integrating a parameter-priority-weighted score, a cluster-compressed redundancy score, and a Z-score-based distance-from-mean score. The distribution of continuous ESp scores was analyzed using an elbow-method approach to identify natural breakpoints separating distinct score ranges. The analysis was performed independently on the upstream regions of *Escherichia coli *(*E. coli*) and *B. bifidum*. As a qualitative validation step, the scoring was applied to a set of *E. coli* K12 upstream regions with known promoter activity, including constitutive promoter sequences from the iGEM catalog^[62]^. The use of *E. coli* K12 enabled a qualitative evaluation of the pipeline by identifying ESp score ranges associated with strong, weak, or absent promoters, as defined and characterized *in vitro*. Similar ranges were identified in *B. bifidum* PRL2010, allowing the assignment of discrete values reflecting the putative propensity for double-strand unwinding: 0.99, 0.66, or 0.33 depending on the degree of this propensity, or 0 otherwise.

At each upstream region, a putative operon value (POV) was assigned as the sum of the presence of hypothetical RPBs (1/0), TFBs (0.5/0), and the discrete value of the ESp. 

### Genetic evolution of *B. bifidum*

Phylogenetic analyses were performed using RAxML^[63]^ on the Roary output for all three genomic datasets. The Roary gene presence-absence matrix for each dataset was integrated with its corresponding core-genome phylogenetic tree to examine patterns of gain and loss of function (GoF/LoF). A Wagner parsimony model was applied to each Roary COG to evaluate GoF/LoF and the statistical significance of each event^[64]^. Subsequently, the influence of the presence of GoF/LoF genes on genome distribution was also evaluated by Principal Coordinates Analysis (PCoA) based on Jaccard distances calculated from the presence matrix of the genes of interest.

### RNA extraction and bioinformatic transcriptomic analysis.

*B. bifidum *PRL2010 was selected as the model strain for transcriptomic profiling due to its extensive genomic and functional characterization, including detailed annotation of the host-glycan degradation genes, and because it is the most documented strain belonging to this species in terms of genetics and interaction with the human gut microbiota^[4]^.

On this basis, transcriptomic data for *B. bifidum* PRL2010 grown on a panel of host-derived glycans representative of the major structural classes of HMO^[65]^ and of the intestinal mucosal environment^[66]^, were retrieved from a previously published study, including 2’-fucosyllactose (2FL), 3’-fucosyllactose (3FL), 3’-sialyllactose (3SL), 6’-sialyllactose (6SL), disialyllacto-N-tetraose (DSLNT), lactosamine, lacto-N-neotetraose (LNnT), lacto-N-tetraose (LNT), and mucin [Supplementary Table 3]. All glycans were used at a final concentration of 1% (wt/vol), as previously described^[2]^. Furthermore, transcriptomic data using GlcNAc were newly generated in the present study.

*B. bifidum* PRL2010 was inoculated into de Man-Rogosa-Sharpe (MRS) (Scharlau Chemie, Barcelona, Spain) medium, where the carbohydrate source was represented by either 1% GlcNAc (wt/vol) (Carbosynth Ltd, UK) or glucose, as a control (Sigma-Aldrich), adjusting the culture to a final optical density at 600 nm (OD600) of 0.2. Cultures were incubated at 37 °C under anaerobic conditions (2.99% H_2_, 17.01% CO_2_, and 80% N) for 8 h. All experiments were performed in triplicate. Following incubation, cultures were centrifuged (7,000 rpm for 15 min) to separate the cell pellet from the supernatant. All aliquots were stored at -80 °C until RNA extraction.

Total RNA from each bacterial culture was isolated as previously described^[67]^. Briefly, cell pellets were initially suspended in 1 mL of QIAZOL lysis reagent (Qiagen, United Kingdom) and transferred into tubes preloaded with 0.8 g of 106 μm glass beads (Sigma-Aldrich). Mechanical cell disruption was performed using a bead beater, with alternating cycles of 2 min of vigorous agitation followed by 2 min of rest on ice. After lysis, samples were centrifuged at 12,000 rpm for 15 min, and the aqueous phase, containing the RNA, was carefully recovered. RNA purification was then completed using the RNeasy Mini Kit (Qiagen, Germany) following the manufacturer’s guidelines.

Total RNA was isolated from cultures at the exponential growth phase (OD_600nm_ between 0.6 and 0.8). RNA quality and integrity were assessed using a TapeStation 2200 (Agilent Technologies, USA), while concentration was determined via a Picodrop microliter spectrophotometer (Picodrop, UK). Whole-transcriptome libraries were prepared using the Illumina Stranded Total RNA Prep, Ligation with Ribo-Zero Plus (Illumina, USA), following the manufacturer’s instructions, and a specific depletion step utilizing the Ribo-Zero Plus Microbiome probe pool to remove ribosomal RNA (rRNA). Fragmented and adapter-ligated cDNA libraries were sequenced on an Illumina platform.

Raw reads were quality-filtered and aligned to the *B. bifidum* PRL2010 reference genome using METAnnotatoX2 RNA-specific tool^[36]^. Quantification of unique reads mapping to Open Reading Frames was performed using HTSeq^[68]^. Differential gene expression analysis between GlcNAc-grown cells and glucose-grown cells was conducted with edgeR v4^[69]^.

### Cultivation of *Bifidobacterium* strains in a chemically defined medium (CDM)

*B. bifidum* PRL2010, *B. bifidum* LMG 11041, *B. bifidum* 184B,* Bifidobacterium callitrichos* DSM 23973, *B. biavatii* DSM 23969, and *B. leontopitheci* LMG 31471 were cultivated in de Man-Rogosa-Sharpe (MRS) medium supplemented with 0.05% L-cysteine hydrochloride in an anaerobic chamber (Concept 400, Ruskinn) at 37 °C. In this context, *B. bifidum* 184B was selected due to the higher ANI divergence from the type strain LMG 11041 and the reference strain PRL2010. After overnight growth, bacterial cultures were diluted, when necessary, to reach an initial OD600 of 0.2, washed in PBS, and resuspended in a previously formulated carbohydrate-free defined medium containing (per liter of distilled water) 4.0 g of sodium acetate; 1.0 g of tri‐ammonium citrate; 2.0 g of KH_2_PO_4_; 2.0 g of K_2_HPO_4_; 0.5 g of MgSO_4_; 0.05 g of MnSO_4_; 0.02 g of FeSO_4_; 0.2 g of CaCl_2_; 20 mg of adenine; 40 mg of xanthine; 0.4 g of cysteine; 0.3 g of aspartic acid; 0.3 g of glutamic acid; 0.2 g of each the following amino acids: alanine, arginine, glycine, histidine, isoleucine, leucine, lysine, methionine, phenylalanine, proline, serine, threonine, tryptophan, tyrosine and valine; 0.5 g of orotic acid; 0.5 mg of p‐aminobenzoic acid; 0.5 mg of folic acid, 2.0 mg of nicotinic acid; 2.0 mg of Ca‐pantothenate; 1.0 mg of biotin; 2.0 mg of pyridoxal; 2.0 mg of riboflavin; and 1.0 mg of vitamin B12 and 0.02 mg of uracil 0.02 mg. Subsequently, the medium was supplemented with either 1% (w/v) glucose (control condition), 1% GlcNAc, or 1% N-acetylgalactosamine (GalNAc). The medium was sterilized by filtration (0.22 μm). Cells were incubated anaerobically at 37°C for 6 h. All cultures were grown in triplicate.

### qPCR-based analysis of *lic*C gene expression

Bacterial RNA was used to analyze the *lic*C gene expression by quantitative real-time PCR (qPCR). Specifically, 500 ng of total RNA from each sample was reverse transcribed to cDNA using the iScript Select cDNA Synthesis Kit (Bio-Rad Laboratories) under the following thermal cycling conditions: 5 min at 25 °C, 30 min at 42 °C, and 5 min at 85 °C. At the end of the process, 25 ng/µL cDNA aliquots were amplified in a total reaction volume of 10 µL using the qPCRBIO SYBR Green Mix Lo-Rox (PCR Biosystems) and the forward and reverse primers (5 pmol each). The primers used for *lic*C amplification were: *lic*C_group2_forward 5’- ACTGCTTCTGCAAGTTCGG -3’ and *lic*C_group2_reverse 5’- GGATGAGCATGATCGGGTT -3’, *lic*C_LMG 31471_forward 5’- GCTCTGGTCGCCTTCTTCA -3’ and *lic*_LMG 31471_reverse 5’- GACGACGATGGCGGTAATC -3’, *lic*C_50T_forward 5’- GGCATCTTCAACCAGACCT -3’ and *lic*C_50T_reverse 5’- CTTGATGCAGGCGGTGTAGA -3’.

Expression levels were normalized relative to housekeeping genes as previously described in different studies^[70,71]^. Real-time PCR was performed using the CFX96 system (Bio-Rad, CA, USA). Fluorescence was monitored at the end of each extension step. Melting curve analysis was performed at the end of each amplification cycle. Data analysis was performed using the relative standard curve method^[72]^. The expression values were calculated as the ratio between the abundance of the target mRNA under each test condition and its abundance in the corresponding glucose-grown control.

## RESULTS

### *B. bifidum *revealed limited intraspecific genetic diversity, with most variation driven by a small subset of African-derived genomes

To comprehensively characterize the *B. bifidum* taxon, an extensive collection of genomic sequences was curated from public repositories, complemented by newly DNA-sequenced genomes of isolated *B. bifidum* strains, and by the assembly of new MAGs. After genome quality control and dereplication using CheckM2 and drep^[38,39]^, the final *B. bifidum* dataset encompassed 1,351 unique high-quality sequences, including 156 genomes from cultured isolates and 1,195 MAGs [Supplementary Table 1]. The inclusion of MAGs enabled exhaustive screening of the species’ genetic diversity, resulting in a closed pangenome [Figure 1A], as previously accomplished for *B. adolescentis* and *B. longum*^[33,34]^.

**Figure 1:**
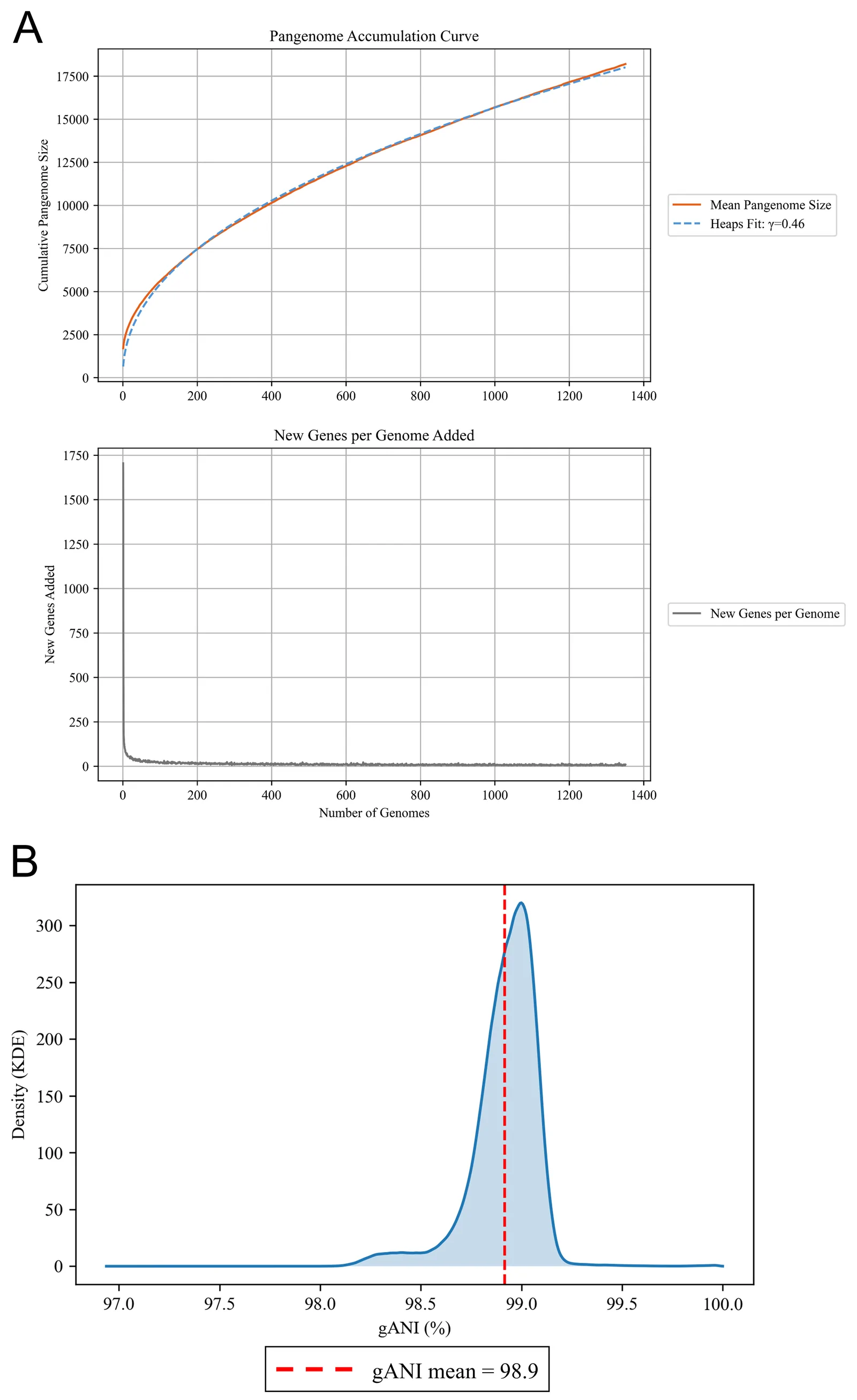
Pangenome architecture of the *B. bifidum* dataset. (A) illustrates the pangenome growth trends, showing variation in pangenome size and the identification of new genes resulting from the addition of 1,351 *B. bifidum* genomes, in the upper and lower graphs, respectively; (B) shows the genetic distance in the *B. bifidum* dataset via a density plot of ANI values obtained from pairwise comparison of genomes. KDE: Kernel density estimation; gANI: genome Average Nucleotide Identity; *B. bifidum*: *Bifidobacterium bifidum.*

In contrast, analysis of the datasets generated in this project revealed that the other HBS pangenomes remain open [Supplementary Figure 1]. Reconstructing the HBS pangenomes enabled a comparative assessment of their genomic features relative to those of *B. bifidum*, providing insights into their genetic diversity. In the comparison among the HBS and PBS [Supplementary Table 2], *B. bifidum* had the smallest genome size and the fewest coding DNA sequences, followed by *B. adolescentis* and *B. pseudocatenulatum*. Overall, the genomic features of *B. bifidum* show mean values closer to those of HBS than PBS, except for GC content, which remains above 60% as in other PBS.

ANI analysis revealed a mean of 98.91 ± 0.16% [Figure 1B], confirming remarkable genetic homogeneity and the absence of subspecies taxonomic divisions, in contrast to other HBS such as *B. catenulatum* and *B. longum* [Supplementary Figure 2]. However, we identified a specific subset of 22 *B. bifidum* genomes, 19 of which were from rural populations in Malawi and Mozambique, showing ANI values below 98.5%. Notably, phylogenomic analysis did not support the presence of a distinct lineage composed of these genomes. In the ANI-based phylogenomic tree, the 22 genomes occupy isolated positions rather than forming a distinct branch [Supplementary Figure 3]. This topology suggests that their divergence does not define a subpopulation shaped by the same selective pressures but rather reflects variation in accessory gene content within this geographical region. This characteristic is consistent with the higher biodiversity observed in non-Westernized settings, where more heterogeneous dietary and environmental exposures may allow a broader range of accessory gene content to persist than is usually observed in genomes from industrialized populations^[73-75]^. Similar patterns, in which non-Westernized or rural cohorts exhibit greater strain-level and accessory genome diversity than Westernized populations, have been reported within the genus *Bifidobacterium*^[34,76]^.

This localized ANI divergence may be due to 46 nearly exclusive clusters of orthologous genes (COGs), 20 of which are completely absent from the rest of the dataset [Supplementary Table 4]. These genes were organized into seven putative genomic islands or functional units, suggesting a specialized gene toolkit for the rural gut environment. Functional annotation revealed that several of these genes encode glycosyltransferases (GT) from the GT1 and GT2 families and components of the Wzy-dependent pathway, which are likely involved in specific cell wall and exopolysaccharide modification. Furthermore, two genomic islands harbored functional domains involved in the degradation and import of fucosylated glycans and complex hexosamines. The limited presence of these functions in this subset of genomes may reflect niche-specific adaptation in non-Westernized populations, despite the overall genomic stability of *B. bifidum*.

### *B. bifidum* possesses the highest mobilome-driven diversity among HBS

The pronounced genetic homogeneity of *B. bifidum*, as indicated by ANI values, is further supported by the lack of significant variation in metabolic gene content across the pangenome of this taxon. Specifically, analysis of the glycobiome provided no evidence for subclusters with distinct carbohydrate-degrading capacities, suggesting a highly conserved metabolic core.

By evaluating the GoF/LoF mechanisms within the dataset, we identified 208 COGs with significant GoF and 245 COGs with significant LoF [Supplementary Table 5], which effectively separated the dataset into four distinct groups [Figure 2A and Supplementary Figure 4]. 

**Figure 2:**
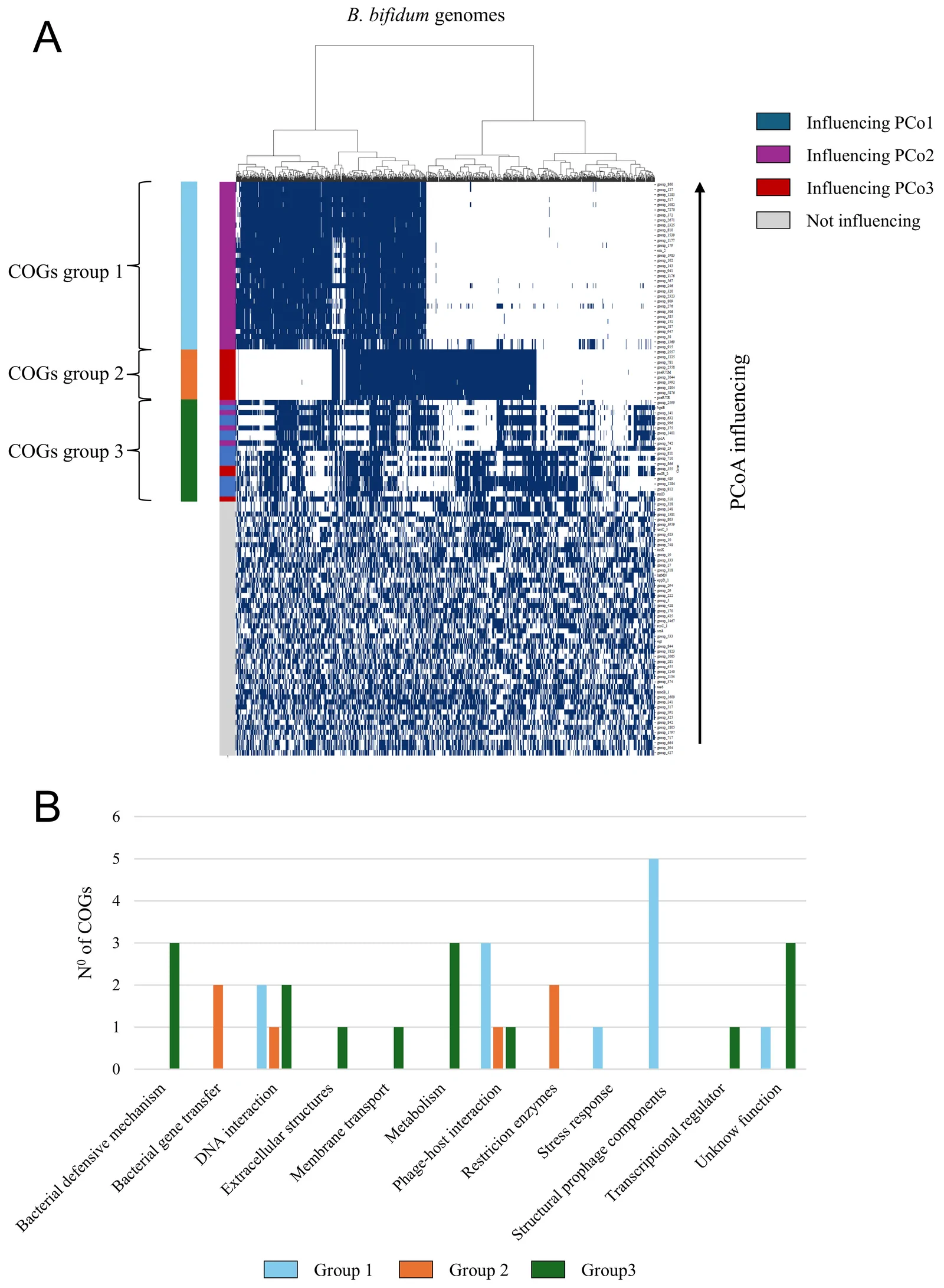
Analysis of the 113 bipartite GoF/LoF COGs in the *B. bifidum* dataset. (A) shows a heatmap of COG presence/absence in the genomes, with the three COG groups highlighted to the left. The upper dendrogram shows the dataset’s genomes redistributed into four subclusters based on the presence of the COGs considered; (B) shows the distribution of the COGs of the three identified groups into different functional groups, based on the PROKKA and Roary annotations and the protein domains identified by InterProScan. COG: Clusters of orthologous gene; PCo: principal coordinate; GoF/LoF: gain and loss of function.

This variation is largely driven by the mobilome (25.4% of COGs), particularly prophage-related elements in COGs group 1, and restriction-modification systems in COGs group 2 [Figure 2B]. In the absence of characteristic plasmids in *B. bifidum*, these clusters likely represent episome-derived elements integrated into the chromosome^[78-81]^.

To assess the distribution of CRISPR-Cas systems across the species, we screened the 1,351 *B. bifidum* genomes using CRISPR-CasFinder. CRISPR loci were identified in 16.95 % of genomes harboring a high-confidence Cas gene cluster, i.e., 84 type I-C, one type class I-E, 139 type class II-A, and five type II-C systems. Type I-C clusters consistently encoded the cas3, cas5, cas8c, cas7, cas4, cas1 and cas2 genes, while class II-A clusters encoded cas9, cas1 and csn2. BLASTN comparison of the predicted spacer repertoire against the NCBI phage genome database detected prophage-derived spacer matches in 195 genomes, 186 of which specifically targeted *Bifidobacterium* phage Bbif-1 (GQ141189), identifying it as the predominant prophage target of CRISPR-Cas-mediated immunity in this species [Supplementary Table 6].

Given the impact of the mobilome on species biodiversity, the propensity of *B. bifidum* for horizontal gene transfer (HGT) was evaluated. Evaluation of the CUB and the GC content of each gene suggested that *B. bifidum* exhibits a different HGT rate than HBS [Figure 3A], potentially reflecting a mechanism conserved across PBS.

**Figure 3:**
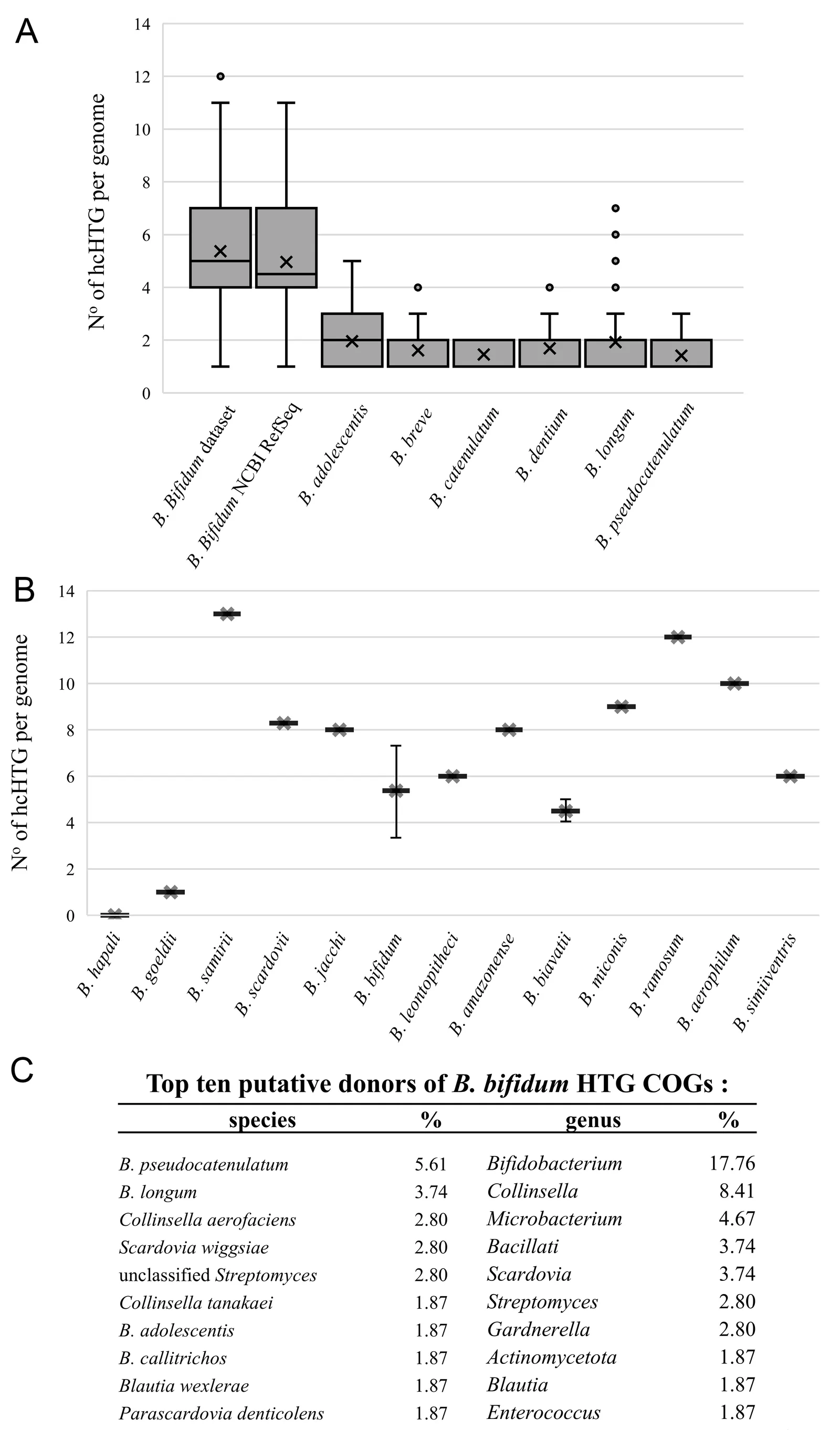
HGT screening across species analyzed. (A) shows the distribution of hcHTG in the different monospecific HBS datasets; (B) shows the number of hcHTG identified in the PBS; (C) shows the putative donors of transferred genetic material at the species level (left) and the genus level (right). hcHTG: Highly confident horizontal transfer gene; HGT: horizontal gene transfer; HBS: human-associated bifidobacterial species; PBS: phylogenetically related *B. bifidum* species.

On average, we identified 5 ± 4 putatively acquired genes in *B. bifidum*, compared to 7 ± 4 in other PBS [Figure 3B and Supplementary Table 7]. Statistical analysis of the hcHTG variance across genomes in the three groups revealed that the internal variability of the *B. bifidum* dataset was more similar to that observed in the PBS than to that in the HBS [Supplementary Figure 5]. Based on amino acid sequence similarities, hypothetical donors include HBS (*B. pseudocatenulatum*, *B. longum*, *B. adolescentis*), *Bifidobacterium callitrichos, *a species associated with the gut microbiota of the non-human primate *Callithrix jacchus*, followed by *Blautia *spp., and *Collinsella* spp. [Figure 3C and Supplementary Table 8].

Results of the intraspecific comparison suggested that *B. bifidum* has reached broad stability in its core genome while retaining sufficient plasticity to acquire accessory genes or variants, particularly in those genomes associated with non-Westernized rural populations.

### Unique metabolic and enzymatic repertoire of *B. bifidum* for host-niche adaptation

Given the highest HGT rate observed in *B. bifidum* compared to HBS, we performed an interspecific comparison with HBS and PBS to identify unique traits acquired during adaptation to the human gut. A comparative analysis of metabolic networks showed that *B. bifidum* was significantly enriched in genetic modules associated with carbohydrate uptake and consumption, energy production, and vitamin metabolism [Supplementary Figure 6].

Large-scale functional screening [Figure 4] identified 17 KEGG functions exclusively present in *B. bifidum* relative to all other HBS.

**Figure 4:**
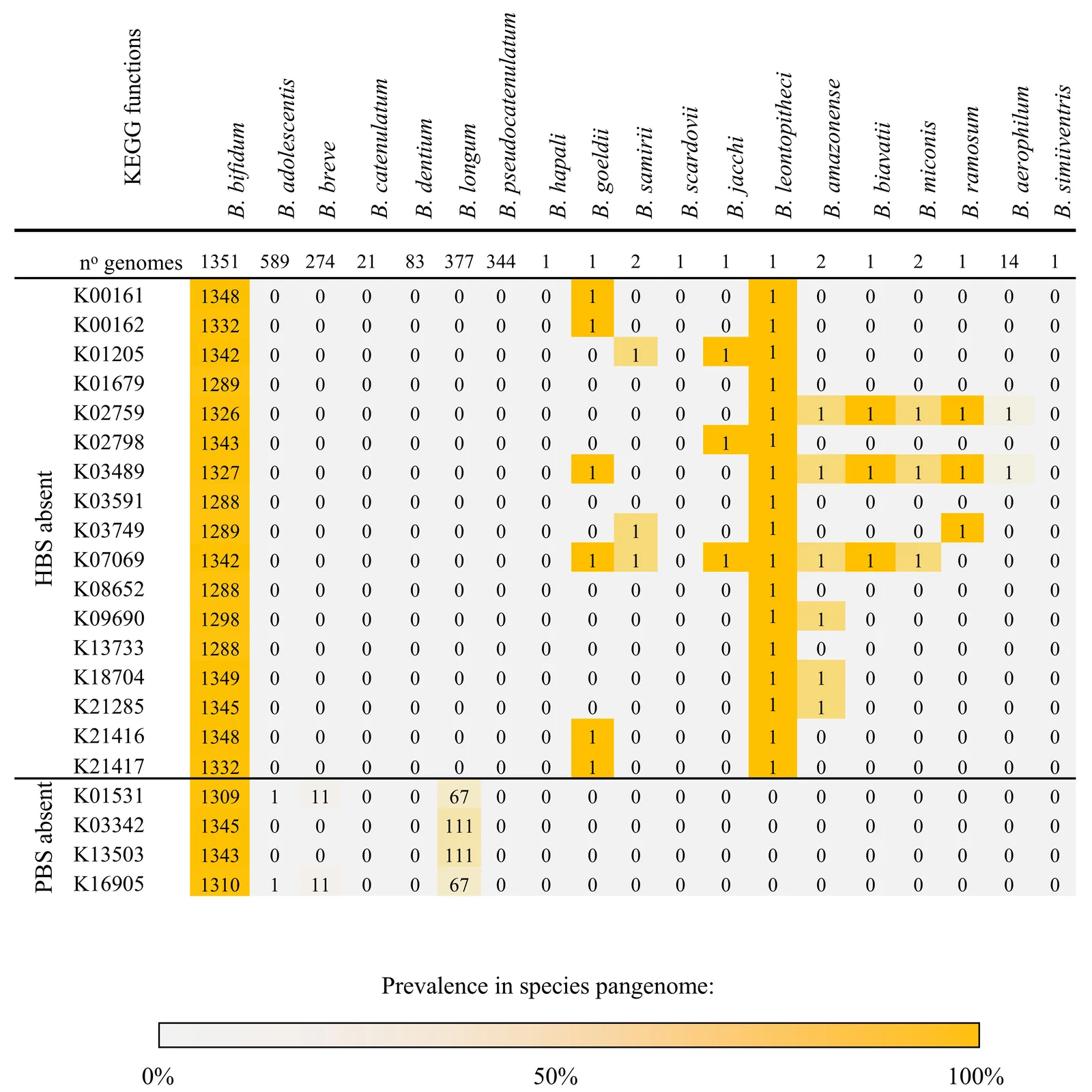
Distribution and prevalence of species-specific functional traits in *B. bifidum*. The heatmap illustrates the prevalence of KEGG orthologs (KOs) belonging to the *B. bifidum* soft-core that are absent or rare in other HBS (top part) and PBS (bottom part). The color intensity and the numbers within each cell represent the total count of genomes per species harboring each specific KO, underscoring the high conservation of these traits across the 1,351 *B. bifidum* genomes. KEGG: Kyoto Encyclopedia of Genes and Genomes; HBS: human-associated bifidobacterial species; PBS: phylogenetically related *B. bifidum* species.

A significant portion of these unique traits was dedicated to cell wall biosynthesis and environmental interaction, including enzymes for teichoic acid synthesis (K18704, K21285), exopolysaccharide transport (K09690), and cell wall modification or anchoring (K03591, K13733, K03749). The analysis also highlighted species-specific metabolic components, including subunits of two different Phosphotransferase Systems (PTS) (K02759, K02798), immunomodulatory proteins (K08652), and transcriptional regulators associated with glycosyl hydrolases (K03489). Furthermore, *B. bifidum* possesses a unique Acetyl-CoA regeneration system encoded by KEGG orthologs K00161, K00162, K21416, and K21417^[82]^.

Consistent with this metabolic profile, *B. bifidum* harbored a highly specialized glycobiome, with a substantial fraction of predicted enzymes to target host glycans. *B. bifidum* dedicates 43% of its 48 highly conserved glycosyl hydrolases (GH) to host-derived glycans, such as HMOs and mucin [Figure 5A and Supplementary Table 9]. 

**Figure 5:**
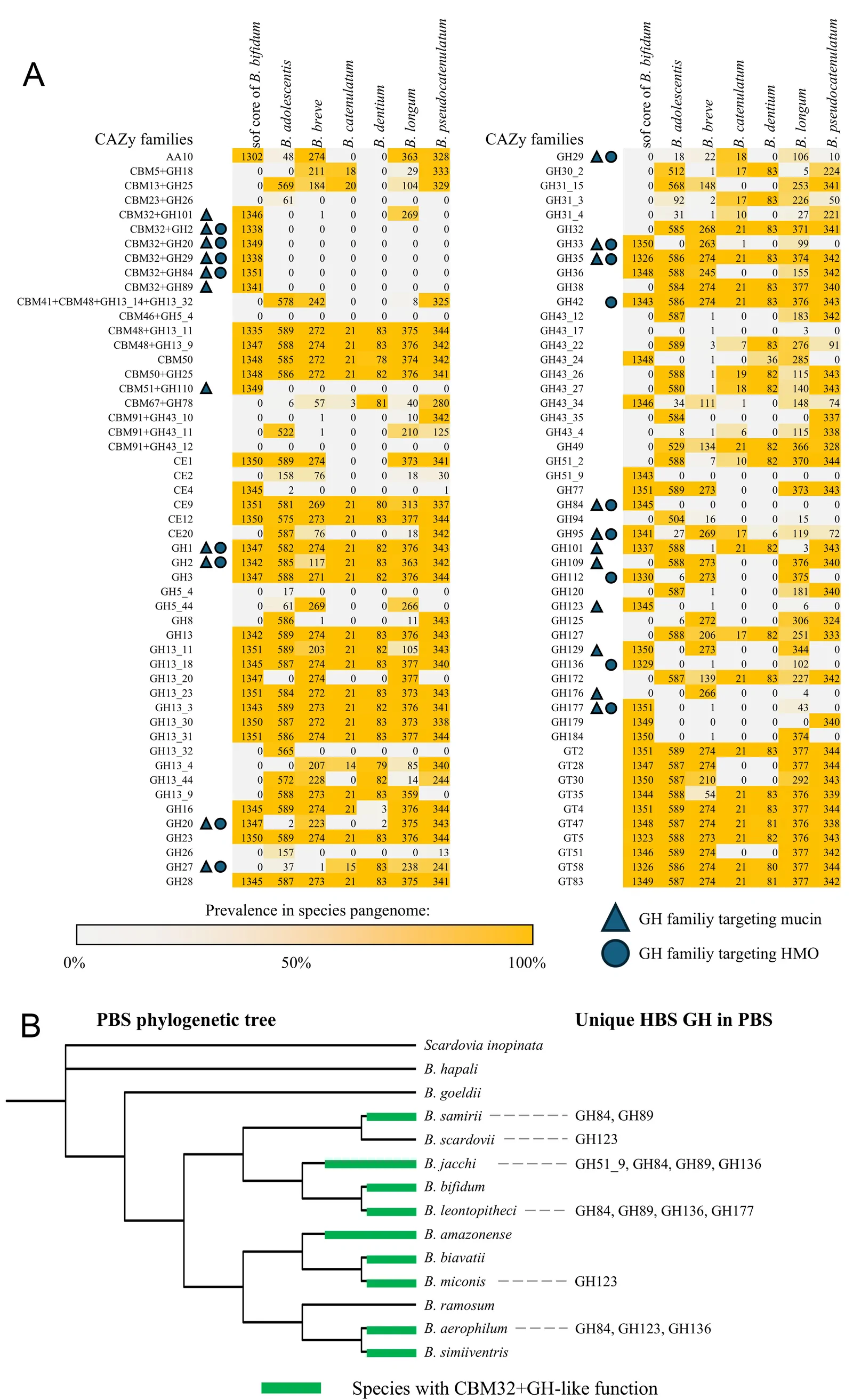
Overview of the *B. bifidum* glycobiome compared to HBS and PBS. (A) shows the heatmap of the presence/absence of the CAZy enzyme families identified in the soft-core genome of *B. bifidum* and in the other HBS. Families with potential enzymatic activity towards carbohydrates present in HMO and the intestinal mucin layer are highlighted by a blue triangle or a blue circle, respectively; (B) shows the presence in PBS of the CAZy enzymatic functionalities absent in the other HBS. The green rectangle reports the PBS with CBM32 associated with different GH families. CAZy: Carbohydrate-Active enZYmes; GH: glycosyl hydrolases; HBS: human-associated bifidobacterial species; PBS: phylogenetically related *B. bifidum* species; HMO: human milk oligosaccharide; CBM32: carbohydrate-binding module 32.

This accounts for 71% of the total host-glycan-targeting GH families identified across the HBS pangenome, significantly exceeding the group average (32 ± 6%). Comparative analysis identified eight GH families absent or rare in other HBS, namely, GH43 subfamily 24, GH51 subfamily 9, GH84, GH89, GH110, GH123, GH136, and GH177, and six unique COGs combining a GH domain with galactose high-affinity carbohydrate-binding module 32 (CBM32)^[83]^. While all these glycobiome features were found with variable degrees in PBS [Figure 5B], *B. bifidum* displays the highest complexity and richness in enzymes dedicated to host-derived substrates. These results suggest that the evolutionary success of *B. bifidum* in colonizing the infant gut relied on a coordinated expansion of its degradative and transport machineries, acting as coupled systems for the simultaneous breakdown and uptake of host-derived glycans.

### Specialized functional islands shape the evolutionary trajectory of *B. bifidum*.

To delineate the unique genetic blueprint of *B. bifidum*, we compared its pangenome with that of the other six HBS, identifying 667 COGs unique to *B. bifidum* soft-core [Supplementary Table 10]. This extensive repertoire of *B. bifidum*-specific COGs is primarily dedicated to cellular metabolism (34.8%), membrane transport (12.8%), and transcriptional regulation (9.7%). Specifically, 25 unique COGs are implicated in the import and processing of human milk sugars^[84]^, while others are associated with host-immune interactions^[85]^ and osmotic and pH regulation^[86]^, i.e., functions that support survival in the infant gut^[23,87,88]^. In addition, 37 COGs harbored unique functional domain annotations within the HBS pangenome. Among these specific protein signatures, in addition to amino acid and cofactor metabolism, redox and transcriptional regulation, there were COGs associated with two PTS systems (K02759, K02798) and teichoic acid biosynthesis (K18704, K21285), previously highlighted in the metabolic comparative analysis.

Genomic organization of the *B. bifidum* reference strain PRL2010^[89]^ using a Putative Operon Value (POV), predicted 40 operons comprising three or more unique COGs [Supplementary Table 11]. Fundamental processes, such as nucleotide and cofactor metabolism, were constitutively expressed under strong promoters (POV from 2.16 to a maximum of 2.49), whereas operons linked to niche adaptation, such as those for cellular response mechanisms to stress, exhibited POV values between 1.83 and 2.16 [Figure 6A]. 

**Figure 6:**
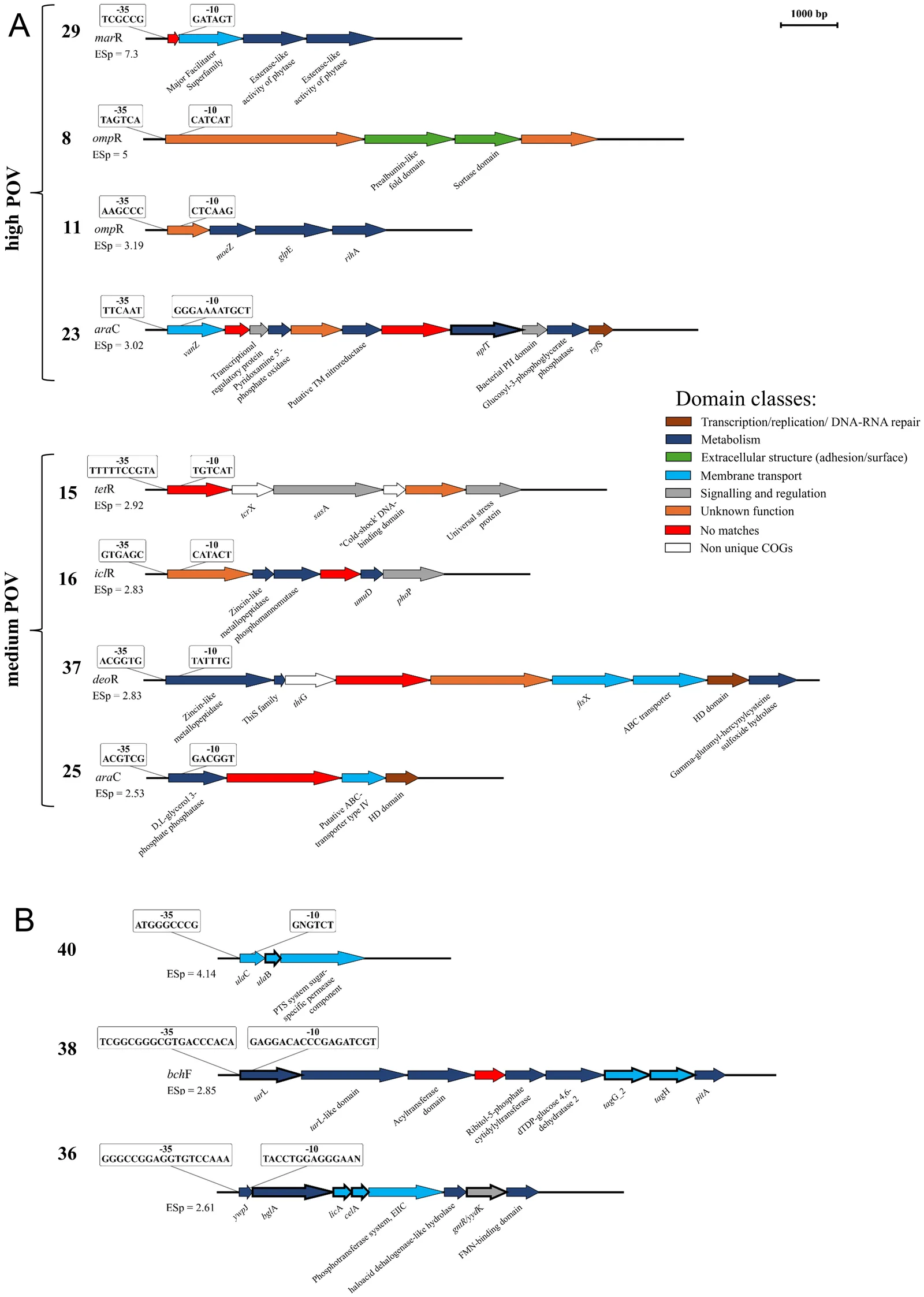
Putative *B. bifidum *operons of unique COGs, sorted by POV value. For each putative operon, the hypothetical RPBf sequences, the matching TFBs, and the energetic-structural property index of the uptake region are reported. For each gene, the orthologous gene in Roary or the protein domain identified by Interproscan is reported. (A) shows operons with medium-to-high POV values; (B) shows operons formed by COGs carrying functional annotations unique to *B. bifidum* compared to HBS. POV: Putative Operon Value; COG: clusters of orthologous gene; TFB: transcription factor binding site; ESp: energetic and structural properties; HBS: human-associated bifidobacterial species; RNB: RNA polymerase binding site.

Of the 37 COGs with unique functional annotations, only those with PTS systems and teichoic acid biosynthesis were arranged in three putative genetic operons [Figure 6B]. Unlike the other putative genetic operons, these exhibit a POV lower than 1, suggesting that their activation responds to specific conditions that allow the transcription of these functional islands.

The distinct evolutionary trajectory of *B. bifidum* is further underscored by the identification of 76 COGs exhibiting significant GoF events relative to both HBS and PBS [Supplementary Table 12]. Notably, Operons 36 and 40, reported in Figure 6B, represent substantial evolutionary acquisitions, encoding specialized PTS systems absent in other HBS. More specifically, operon 40 encodes a PTS system whose three subunits exhibit high amino acid identity (up to 96.7%) with the closest PBS, *B. jacchi*, and *B. leontopitheci*, as well as high identity to *Bombiscardovia coagulans* (77.5%) and *Gardnerella* spp. (83%-85%). This high level of conservation across genera suggests gene acquisition from primate-associated bifidobacteria and related *Bifidobacteriaceae*. Likewise, operon 36 encodes a 4.A.3 family PTS^[90]^, a GH1 6-phospho-beta-glucosidase (*bgl*A), and a *gnt*R/*yyd*K-like regulator [Figure 6B]. This gene cluster shares 80.4% sequence identity with *B. callitrichos* and exhibits broader homology with more distantly related anaerobic genera, such as *Clostridium* (57.6%), *Eubacterium* (57.3%), and *Carnobacterium* (59.3%). By comparing the amino acid sequences encoded by the genes of the PTS systems with those of the PBS, we obtained a division of the species cluster. Specifically, i.e., *B. leontopitheci*, *B. amazonense*, *B. biavatii*, *B. miconis*, *B. ramosum*, and *B. aerophilum*, showed significant matches with an average identity of 53.8 ± 22.2, whereas no significant matches were identified for the remaining species. These patterns suggest a more complex or ancestral acquisition event. Overall, these results indicate that *B. bifidum* has enhanced its metabolic repertoire by acquiring high-affinity transport systems from a wide phylogenetic spectrum, thereby gaining a significant fitness advantage in colonizing the human intestinal environment.

### *In vitro* characterization of unique *B. bifidum* PTS and GH associated with host-derived glycans

To validate the functional relevance of the identified evolutionary gains, we analyzed the transcriptional profile of *B. bifidum* PRL2010 grown on different host-glycan substrates, such as^[2]^ [Supplementary Table 3]. In addition, we conducted targeted follow-up transcriptomic assays to support and extend earlier observations. Our analysis revealed a coordinated activation of species-specific genes [Figure 7], including unique PTS systems and six GHs carrying an associated CBM32 module, which were absent in the other HBS [Figure 5A].

**Figure 7:**
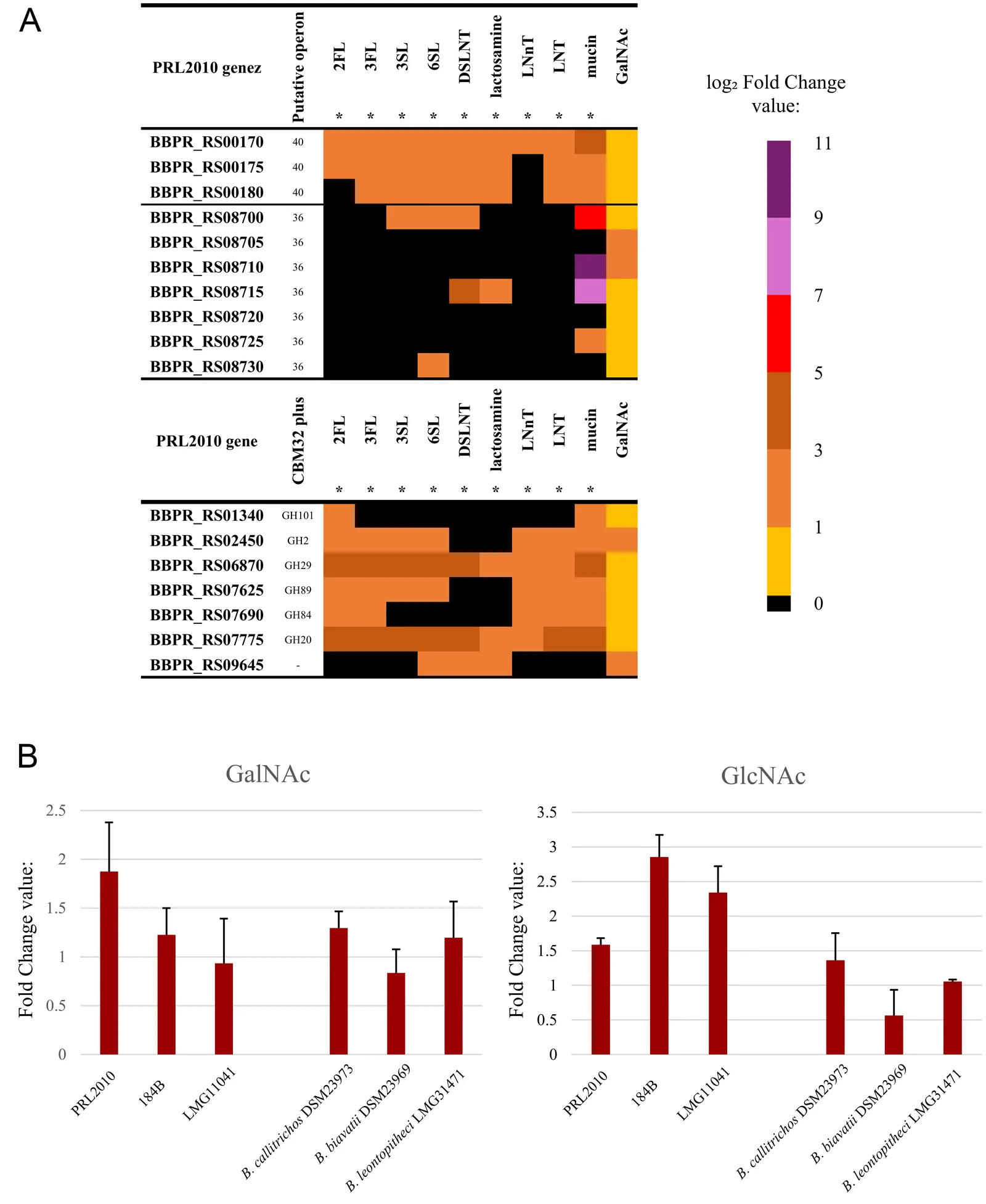
Transcriptional response of species-specific PTS and CBM32-containing GH in *B. bifidum *PRL2010. (A) the heatmap shows differential gene expression (log_2_FC). The upper panel displays transcriptional activation of the species-specific PTS systems encoded by operons 36 and 40, while the lower panel shows induction patterns of the six GH genes harboring CBM32 modules. Color intensity represents the log_2_FC values relative to glucose (used as reference carbon source), with black cells indicating basal or non-significant expression (log_2_FC < 1). Substrates marked with an asterisk (*) indicate transcriptomic data integrated from a previous study^[2]^, whereas the data for GlcNAc were generated in this study; (B) the histograms show RT-qPCR-based fold-change values for the *lic*C homolog in six microorganisms, *i.e.*, *B. bifidum* PRL2010, *B. bifidum *184B, *B. bifidum *LMG 11041, *B. callitrichos* DSM 23973, *B. biavatii* DSM 23969, and *B. leontopitheci* LMG 31471. For each strain, fold-change values indicate *lic*C expression during growth on GlcNAc and GalNAc relative to the glucose-grown condition used as reference. PTS: Phosphotransferase systems; CBM32: carbohydrate-binding module 32; GH: glycosyl hydrolases; FC: foldchange; GlcNAc: N-acetylglucosamine; GalNAc: N-acetylgalactosamine; 2’-fucosyllactose; 3FL: 3’-fucosyllactose; 3SL: 3’-sialyllactose; 6SL: 6’-sialyllactose; DSLNT: disialyllacto-N-tetraose; LNnt: lactosamine, lacto-N-neotetraose; LNT: lacto-N-tetraose.

Specifically, the PTS system encoded by operon 36 and several CBM32-containing GHs showed a significant transcriptional response to mucin compared with glucose. Two genes encoding subunits of the latter PTS exhibited the strongest induction (log_2_FC > 7), which correlated with significant up-regulation of mucin-targeting enzymes, such as CBM32-containing GH29 and GH20 [Figure 7]. This coordinated activation was also observed when *B. bifidum* PRL2010 was grown on GlcNAc as the sole carbon source. In this condition, only 20 genes of *B. bifidum* PRL2010 were significantly up-regulated (log_2_FC > 1), including the two PTS subunits of operon 36. Notably, the core catabolic enzymes *nag*A and *nag*B remained constitutively active in both glucose and GlcNAc, confirming that the pathway is functionally operative for direct metabolism rather than solely for cell wall structural biosynthesis.

A similar trend of coordinated response was observed for the second species-specific PTS system encoded by operon 40 and the broader CBM32-containing GH repertoire in response to HMOs. The PTS system encoded by operon 40 behaved differently from the mucin-specific PTS of operon 36, showing a broader and more consistent expression pattern across the different host-glycans tested. This effect was most evident with fucosylated and sialylated HMOs, while no significant overexpression was observed on GlcNAc. We observed a similar behavior in several CBM32-containing GHs [Figure 7A], which were strongly induced by 2FL, 3FL, and LNT, suggesting a key role in the utilization of human milk glycans. 

To validate transcriptional induction of the species-specific PTS machinery, we performed RT-qPCR analysis of the *lic*C homolog across three *B. bifidum* strains (PRL2010, 184B, and LMG 11041) and three PBS (*B. callitrichos* DSM 23973, *B. biavatii* DSM 23969, *B. leontopitheci* LMG 31471) grown on GlcNAc or GalNAc. Each condition, including the glucose reference, was assayed in triplicate. All three *B. bifidum* strains showed consistent upregulation of the *lic*C homolog on GlcNAc when normalized on the glucose condition (FCs from 1.6 to 2.9), whereas induction was weak or absent in the PBS taxa tested (FCs from 0.6 to 1.4), supporting a *B. bifidum*-specific transcriptional response to this substrate. In contrast, induction by GalNAc was modest and inconsistent across all strains and species tested (FC ranging from 0.8 to 1.9), indicating that this substrate is not a strong inducer of the *lic*C homolog.

Taken together, these findings demonstrate that *B. bifidum* possesses a specialized, coordinated genetic machinery and unique PTS transporters that enable efficient exploitation of the diverse glycan landscape of the neonatal gut.

## DISCUSSION

In this study, we reconstructed the pangenome of *B. bifidum* using a dataset of 1,351 unique high-quality genomes. This large collection allowed us to better define the genetic landscape of this important neonatal symbiont. By incorporating a large number of MAGs, following an approach previously applied to *B. adolescentis* and *B. longum*^[33,34]^, this study provides a robust framework allowing genomic investigation within a closed *B. bifidum *pangenome. Previous comparative genomics studies of this species, limited to smaller datasets of cultured isolates from Westernized populations, consistently reported an open pangenome structure^[29-32]^. Our approach is consistent with the most recent methodologies for in-depth characterization of microbial species diversity, as in previous studies^[91-93]^. To fully capture a species’ genetic diversity, datasets must be sufficiently large and encompass distinct geographical regions. Indeed, geographical origin is a major driver of intraspecific diversity, shaping host traits from diet to antibiotic exposure^[94-97]^.

The saturation of the pangenome indicates that our current dataset, expanded by the inclusion of MAGs, likely captures most of the species’ genetic biodiversity. This is consistent with the high average ANI across genomes (98.9%), indicating a strong level of genetic homogeneity. Nevertheless, a small subset of 22 genomes, mostly from rural populations in Malawi and Mozambique, generates an outlier tail in the ANI distribution. The genomes of these strains exhibit a localized divergence (ANI < 98.5%) and harbor nearly exclusive genetic clusters, including specific glycosyltransferases and fucosidase variants.> Several environmental factors may have influenced the genomic divergence of this subcluster. The HMO composition in the milk of African mothers appears to differ from that of mothers from Westernized cohorts, with a lower content of fucosylated HMO^[98,99]^, altering the availability of a useful substrate for *B. bifidum*, hypothetically favoring strains harboring the specific fucosidase gene variants. The post-weaning diet, typically rich in high-fiber plant foods in sub-Saharan Africa, may also play an important role in modulating the microbiota and selecting for specific strains^[100,101]^. Finally, the differential exposure to antibiotics between Westernized and non-Westernized cohorts can also be considered a selective force that allows the persistence of different strains in populations with low antibiotic exposure^[102,103]^. This suggests that while the pangenome appears closed, a modest but significant biodiversity persists in some isolated regions, likely reflecting niche-specific adaptations to non-Westernized lifestyles. The results redefine *B. bifidum* as the most specialized HBS in terms of host-glycan utilization.

This finding provides a new quantitative perspective that adds to the results of previous studies, which have focused more on descriptive aspects in *B. bifidum*^[29,104]^ and other HBS^[105-107]^. The analysis and comparison of the highly conserved part of the glycobiome of *B. bifidum* with that of other HBS allowed us to define qualitative indices that represent the degree of adaptation to host glycans in HBS. Metagenomic analyses have also highlighted the importance of genes related to human glycan degradation in the early development of the gut microbiota^[108]^. Our results show that 43% of the conserved glycobiome of *B. bifidum* is specifically associated with the degradation of HMOs and mucins. This taxon accounts for 71% of all GH families that target host glycans identified across the entire HBS pangenome, exceeding the group average (32% ± 6%). Furthermore, the identification of eight GH families absent or poorly represented in all other HBS, and six unique COGs that combine GH domains with high-affinity CBM32 modules, defines a highly conserved, species-specific enzymatic signature across the entire closed pangenome, adapted to the ecological niche.

This evolutionary process was characterized by 76 significant gain-of-function events, including the acquisition of specialized functional islands. Our transcriptomic data confirm that these acquisitions, specifically unique PTS systems and species-specific GHs harboring CBM32 domains, act as a coordinated machinery for the efficient uptake and breakdown of mucin and HMOs. The strong transcriptional induction of these systems by host-glycans highlights the high-affinity adaptation of *B. bifidum* to the human gut. These metabolic toolkits, likely acquired from different primate-associated bifidobacteria or other gut-associated taxa, confer on *B. bifidum* a significant fitness advantage during early colonization, enabling it to rapidly exploit the nutrient-rich host niche. Targeted RT-qPCR validation across multiple *B. bifidum* strains and some PBS confirmed that induction of the *lic*C homolog upon GlcNAc exposure is a consistent, *B. bifidum*-specific trait, whereas induction by GalNAc was weak and inconsistent across *B. bifidum *strains and PBS species. This strain-level consistency for GlcNAc, contrasted with the limited response observed in PBS taxa, supports the view that this PTS represents a genuine, conserved adaptation of the species rather than a strain-specific or PRL2010-specific artifact.

Despite genomic stability and the closed nature of its pangenome, *B. bifidum* exhibits a high rate of HGT, comparable to that of other PBS. This suggests that the evolutionary trajectory of *B. bifidum* has been shaped by continuous genetic exchange with other microbiota-associated genera, including *Blautia* and *Collinsella*. Most of the intraspecific variability was concentrated in the mobilome, particularly in genes involved in phage-host interactions and in bacterial new-gene acquisition systems. In contrast, the metabolic core remained largely conserved. This pattern indicates that core metabolic functions remain stable, whereas variability is mainly associated with adaptive and defense-related mechanisms in the neonatal niche. Taken together, our findings support the view of *B. bifidum* as a highly specialized taxon. The genomic framework generated here may help guide the identification of candidate functions relevant to understanding how *B. bifidum* colonizes the host intestinal mucosa and persists within the human gut ecosystem, while providing a rationale for targeted *in vitro* experimental validation.

### Limitation

The extensive addition of MAGs enabled the pangenome to approach closure and allowed us to represent intraspecific variability associated with specific geographic areas. Nevertheless, specific regions, such as South America, remain underrepresented due to the limited availability of gut-derived genomes from unexplored parts of the globe. In addition, because host metadata, such as age and sex, were not publicly available for most of the MAGs, we couldn’t identify a clear, strong correlation between genomic features and host characteristics. Genome-wide transcriptomic profiling was performed on a single strain of *B. bifidum* PRL2010 under standard laboratory conditions. Given the intraspecific divergence identified in this study, this profiling may not fully capture the transcriptional landscape of the whole species. RT-qPCR validation of the *lic*C homolog partially mitigated this limitation by extending the analysis to additional *B. bifidum* strains and PBS taxa, but a genome-wide transcriptomic characterization of a broader strain panel, particularly of the most divergent genomic subset identified, would be needed to fully resolve the extent of intraspecific transcriptional variability. Finally, in the context of a large-scale MAG-based approach, residual assembly artifacts cannot be entirely excluded despite the application of stringent quality-filtering criteria.

In conclusion, this study provides the most comprehensive genomic-scale view of *B. bifidum* to date, revealing limited intraspecific genomic diversity alongside a marked functional specialization toward the human gut environment. Comparative genomic and transcriptomic analyses uncovered a pronounced specialization toward host-derived glycan utilization, including an expanded set of glycoside hydrolases and unique phosphotransferase systems that likely contribute to the ecological success of this species in early life. Despite its overall genomic stability, the identification of localized divergence in strains from non-Westernized populations highlights the persistence of niche-specific evolutionary adaptations. Collectively, these findings define *B. bifidum* as a metabolically specialized cornerstone of the neonatal gut microbiota and provide a robust foundation for future studies on its evolutionary dynamics and host-associated functions.

## DECLARATIONS

### Availability of data and materials

All the new genome sequences from isolated cultures have been deposited and are available in NCBI under the project PRJNA1429307. Genomes assembled from metagenomic samples have been deposited in Zenodo under the identification number 19249701. All publicly available metagenomic datasets supporting the findings of this study can be accessed using the accession code reported in the Supplementary Materials. Transcriptional profiles of *B. bifidum* PRL2010 growth on different glycans from a previous strain-host correlation study^[2]^ were analyzed. The transcriptional profile of growth on GlcNAc is reported in Supplementary Table 11. All the custom scripts used in this study have been deposited in the GitHub repository https://github.com/es95e.

### Copyright

© The Author(s) 2026.

## References

[1] Milani C, Lugli GA, Duranti S (2014). Genomic encyclopedia of type strains of the genus bifidobacterium. Appl Environ Microbiol.

[2] Duranti S, Lugli GA, Milani C (2019). *Bifidobacterium bifidum* and the infant gut microbiota: an intriguing case of microbe-host co-evolution. Environ Microbiol.

[3] Tojo R, Suárez A, Clemente MG (2014). Intestinal microbiota in health and disease: role of bifidobacteria in gut homeostasis. World J Gastroenterol.

[4] Turroni F, Duranti S, Milani C, Lugli GA, van Sinderen D, Ventura M (2019). *Bifidobacterium bifidum*: a key member of the early human gut microbiota. Microorganisms.

[5] Anania C, Matys V, Marra S (2025). Effect of supplementation with a specific probiotic (*Bifidobacterium bifidum* PRL2010) in pregnancy for the prevention of atopic dermatitis in children: preliminary results of a randomized trial. Nutrients.

[6] Zhao M, Ma Q, Meng Y (2026). Protective effects of *Bifidobacterium bifidum* strains with IgA-potentiating activity against ETEC infection in weanling mice. Probiotics Antimicrob Proteins.

[7] Shiver AL, Sun J, Culver R (2025). Genome-scale resources in the infant gut symbiont Bifidobacterium breve reveal genetic determinants of colonization and host-microbe interactions. Cell.

[8] Freitas AC, Li G, Shawon J (2025). Abundance of *Bifidobacterium* species in the infant gut microbiota and associations with maternal-infant characteristics in Dhaka, Bangladesh. mSphere.

[9] Ladeira R, Tap J, Derrien M (2023). Exploring Bifidobacterium species community and functional variations with human gut microbiome structure and health beyond infancy. Microbiome Res Rep.

[10] Pasolli E, Mauriello IE, Avagliano M, Cavaliere S, De Filippis F, Ercolini D (2024). Bifidobacteriaceae diversity in the human microbiome from a large-scale genome-wide analysis. Cell Rep.

[11] Niu MM, Li Y, Ma B, Meng XC (2026). Bifidobacterium bifidum alleviate intestinal barrier damage by regulating the intestinal flora and colonic mucin O-glycan structural patterns. J Agric Food Chem.

[12] Segui-Perez C, Huang LZX, Paganelli FL, Lievens E, Strijbis K (2025). Probiotic *Bifidobacterium bifidum* strains desialylate MUC13 and increase intestinal epithelial barrier function. Sci Rep.

[13] Onodera H, Sato Y, Komatsu Y, Yamashita M, Watanabe Y, Kokubo T (2025). HMOs induce butyrate production of *Faecalibacterium prausnitzii* via cross-feeding by *Bifidobacterium bifidum* with different mechanisms for HMO types. Microorganisms.

[14] Wang X, Zhang G, Jiang H, Sun J, Mao X (2025). Structural diversity of human milk oligosaccharides mediates *Bifidobacterium bifidum*-induced adhesion to intestinal epithelial cells. J Agric Food Chem.

[15] Kim YT, Huang YP, Ozturk G (2025). Characterization of *Bifidobacterium bifidum* growth and metabolism on whey protein phospholipid concentrate. J Dairy Sci.

[16] Qian X, Gu Z, Tang X (2025). Characterization of peptide utilization *in vitro* by *Bifidobacterium bifidum*: insights from computer simulations and verification to enhance nitrogen source utilization. Food Sci Nutr.

[17] Ferrario C, Duranti S, Milani C (2015). Exploring amino acid auxotrophy in *Bifidobacterium bifidum* PRL2010. Front Microbiol.

[18] Ku S, Park MS, Ji GE, You HJ (2016). Review on *Bifidobacterium bifidum* BGN4: functionality and nutraceutical applications as a probiotic microorganism. Int J Mol Sci.

[19] Yue Y, Wang Y, Han Y (2023). Genome analysis of *Bifidobacterium bifidum* E3, structural characteristics, and antioxidant properties of exopolysaccharides. Foods.

[20] Yu J, An N, Cui Y, Zhang L (2025). Structural characterization and immunomodulatory effects of *Bifidobacterium bifidum* biofilms enriched with extracellular polysaccharides and surface proteins. Int J Biol Macromol.

[21] Abdulqadir R, Al-Sadi R, Gupta Y, Rawat M, Ma T (2026). Probiotic bacteria *Bifidobacterium bifidum* upregulation of intestinal epithelial tight junction barrier is mediated by TLR-2/TLR-6 receptor complex activation of occludin gene. NPJ Biofilms Microbiomes.

[22] Turroni F, Serafini F, Foroni E (2013). Role of sortase-dependent pili of *Bifidobacterium bifidum* PRL2010 in modulating bacterium-host interactions. Proc Natl Acad Sci U S A.

[23] Longhi G, Vergna LM, Lugli GA (2025). The role of teichoic acids of bifidobacteria in driving the interaction with the human host. Front Microbiol.

[24] Shin J, Barrangou R (2026). Occurrence and applications of CRISPR-Cas systems in bifidobacteria. Appl Environ Microbiol.

[25] Lugli GA, Alessandri G, Milani C (2020). Evolutionary development and co-phylogeny of primate-associated bifidobacteria. Environ Microbiol.

[26] Lugli GA, Calvete-Torre I, Alessandri G (2021). Phylogenetic classification of ten novel species belonging to the genus Bifidobacterium comprising *B. phasiani* sp. nov., *B. pongonis* sp. nov., *B. saguinibicoloris* sp. nov., *B. colobi* sp. nov., *B. simiiventris* sp. nov., *B. santillanense* sp. nov., *B. miconis* sp. nov., *B. amazonense* sp. nov., *B. pluvialisilvae* sp. nov., and *B. miconisargentati* sp. nov. Syst Appl Microbiol.

[27] Modrackova N, Stovicek A, Burtscher J (2021). The bifidobacterial distribution in the microbiome of captive primates reflects parvorder and feed specialization of the host. Sci Rep.

[28] Michelini S, Modesto M, Filippini G (2016). *Bifidobacterium aerophilum* sp. nov., *Bifidobacterium avesanii* sp. nov. and *Bifidobacterium ramosum* sp. nov.: three novel taxa from the faeces of cotton-top tamarin (Saguinus oedipus L.). Syst Appl Microbiol.

[29] Abdelhamid AG, El-Dougdoug NK (2021). Comparative genomics of the gut commensal *Bifidobacterium bifidum* reveals adaptation to carbohydrate utilization. Biochem Biophys Res Commun.

[30] Lu W, Pei Z, Zang M (2021). Comparative genomic analysis of *Bifidobacterium bifidum* strains isolated from different niches. Genes (Basel).

[31] Wei X, Yu L, Zhang C (2023). Genetic-phenotype analysis of *Bifidobacterium bifidum* and its glycoside hydrolase gene distribution at different age groups. Foods.

[32] Li M, Yu J, Li W, Wu Q, Sun J, Sun Z Comparative genomic analysis revealed that dietary habits affected the adaptation of *Bifidobacterium bifidum* to the intestinal tract in different geographic populations. *Food Sci Hum Wellness *2024;13:3006-17.

[33] Selleri E, Tarracchini C, Petraro S (2026). Assessment of genome evolution in Bifidobacterium adolescentis indicates genetic adaptation to the human gut. mSystems.

[34] Shao Y, Wang S, Gichuki BM (2026). Genomic atlas of Bifidobacterium infantis and *B. longum* informs infant probiotic design. Cell.

[35] Lugli GA, Milani C, Mancabelli L, van Sinderen D, Ventura M (2016). MEGAnnotator: a user-friendly pipeline for microbial genomes assembly and annotation. FEMS Microbiol Lett.

[36] Milani C, Lugli GA, Fontana F (2021). METAnnotatorX2: a comprehensive tool for deep and shallow metagenomic data set analyses. mSystems.

[37] Prjibelski A, Antipov D, Meleshko D, Lapidus A, Korobeynikov A (2020). Using SPAdes *de novo* assembler. Curr Protoc Bioinformatics.

[38] Chklovski A, Parks DH, Woodcroft BJ, Tyson GW (2023). CheckM2: a rapid, scalable and accurate tool for assessing microbial genome quality using machine learning. Nat Methods.

[39] Olm MR, Brown CT, Brooks B, Banfield JF (2017). dRep: a tool for fast and accurate genomic comparisons that enables improved genome recovery from metagenomes through de-replication. ISME J.

[40] O'Leary NA, Wright MW, Brister JR (2016). Reference sequence (RefSeq) database at NCBI: current status, taxonomic expansion, and functional annotation. Nucleic Acids Res.

[41] Majeed A, Sharma V, Ul Rehman W (2026). Comprehensive codon usage analysis across diverse plant lineages. Biochem Genet.

[42] Cook R, Brown N, Redgwell T (2021). INfrastructure for a PHAge REference Database: identification of large-scale biases in the current collection of cultured phage genomes. Phage (New Rochelle).

[43] Kanehisa M, Furumichi M, Sato Y, Kawashima M, Ishiguro-Watanabe M (2023). KEGG for taxonomy-based analysis of pathways and genomes. Nucleic Acids Res.

[44] Cantalapiedra CP, Hernández-Plaza A, Letunic I, Bork P, Huerta-Cepas J (2021). eggNOG-mapper v2: functional annotation, orthology assignments, and domain prediction at the metagenomic scale. Mol Biol Evol.

[45] Palù M, Basile A, Zampieri G (2022). KEMET - a python tool for KEGG Module evaluation and microbial genome annotation expansion. Comput Struct Biotechnol J.

[46] Zheng J, Ge Q, Yan Y, Zhang X, Huang L, Yin Y (2023). dbCAN3: automated carbohydrate-active enzyme and substrate annotation. Nucleic Acids Res.

[47] Cantarel BL, Coutinho PM, Rancurel C, Bernard T, Lombard V, Henrissat B (2009). The Carbohydrate-Active EnZymes database (CAZy): an expert resource for Glycogenomics. Nucleic Acids Res.

[48] Seemann T (2014). Prokka: rapid prokaryotic genome annotation. Bioinformatics.

[49] Sitto F, Battistuzzi FU (2020). Estimating pangenomes with Roary. Mol Biol Evol.

[50] Jones P, Binns D, Chang HY (2014). InterProScan 5: genome-scale protein function classification. Bioinformatics.

[51] Finn RD, Bateman A, Clements J (2014). Pfam: the protein families database. Nucleic Acids Res.

[52] Bottacini F, Zomer A, Milani C (2017). Global transcriptional landscape and promoter mapping of the gut commensal *Bifidobacterium breve* UCC2003. BMC Genomics.

[53] Kozakai T, Izumi A, Horigome A (2020). Structure of a core promoter in *Bifidobacterium longum* NCC2705. J Bacteriol.

[54] Sun Z, Westermann C, Yuan J, Riedel CU (2014). Experimental determination and characterization of the gap promoter of *Bifidobacterium bifidum* S17. Bioengineered.

[55] Sakanaka M, Tamai S, Hirayama Y (2014). Functional analysis of bifidobacterial promoters in *Bifidobacterium longum *and *Escherichia coli* using the α-galactosidase gene as a reporter. J Biosci Bioeng.

[56] Longhi G, Petraro S, Milani C (2025). Genetic characterisation of the *upp* gene in *Bifidobacterium bifidum* PRL2010. Microb Biotechnol.

[57] https://bacregdb.bsu.by/.

[58] Olson WK, Bansal M, Burley SK (2001). A standard reference frame for the description of nucleic acid base-pair geometry. J Mol Biol.

[59] (1998). SantaLucia J Jr. A unified view of polymer, dumbbell, and oligonucleotide DNA nearest-neighbor thermodynamics. Proc Natl Acad Sci U S A.

[60] Young RT, Czapla L, Wefers ZO, Cohen BM, Olson WK (2022). Revisiting DNA sequence-dependent deformability in high-resolution structures: effects of flanking base pairs on dinucleotide morphology and global chain configuration. Life (Basel).

[61] Friedel M, Nikolajewa S, Sühnel J, Wilhelm T (2009). DiProGB: the dinucleotide properties genome browser. Bioinformatics.

[62] http://parts.igem.org/Escherichia_coli.

[63] Stamatakis A (2014). RAxML version 8: a tool for phylogenetic analysis and post-analysis of large phylogenies. Bioinformatics.

[64] Konno N, Iwasaki W (2023). Machine learning enables prediction of metabolic system evolution in bacteria. Sci Adv.

[65] Thurl S, Munzert M, Boehm G, Matthews C, Stahl B (2017). Systematic review of the concentrations of oligosaccharides in human milk. Nutr Rev.

[66] Zúñiga M, Monedero V, Yebra MJ (2018). Utilization of host-derived glycans by intestinal lactobacillus and bifidobacterium species. Front Microbiol.

[67] Turroni F, Milani C, Duranti S (2016). Deciphering bifidobacterial-mediated metabolic interactions and their impact on gut microbiota by a multi-omics approach. ISME J.

[68] Anders S, Pyl PT, Huber W (2015). HTSeq - a Python framework to work with high-throughput sequencing data. Bioinformatics.

[69] Chen Y, Chen L, Lun ATL, Baldoni PL, Smyth GK (2025). edgeR v4: powerful differential analysis of sequencing data with expanded functionality and improved support for small counts and larger datasets. Nucleic Acids Res.

[70] Stenico V, Baffoni L, Gaggìa F, Biavati B (2014). Validation of candidate reference genes in Bifidobacterium adolescentis for gene expression normalization. Anaerobe.

[71] Bustin SA, Benes V, Nolan T, Pfaffl MW (2005). Quantitative real-time RT-PCR--a perspective. J Mol Endocrinol.

[72] Liu Y, Zhong W, Feng S (2023). Identification of new reference genes for colony counting by reverse-transcription quantitative PCR in *Bifidobacterium animalis*. J Dairy Sci.

[73] Kryazhimskiy S, Plotkin JB (2008). The population genetics of dN/dS. PLoS Genet.

[74] Hansen MEB, Rubel MA, Bailey AG (2019). Population structure of human gut bacteria in a diverse cohort from rural Tanzania and Botswana. Genome Biol.

[75] Mah JC, Lohmueller KE, Garud NR (2025). Inference of the demographic histories and selective effects of human gut commensal microbiota over the course of human history. Mol Biol Evol.

[76] Fu P, Qi H, Liu W (2025). Comparative genomics and adaptive evolution of *Bifidobacterium adolescentis* in geographically distinct human gut populations. Foods.

[77] Bottacini F, O’Connell Motherway M, Casey E (2015). Discovery of a conjugative megaplasmid in *Bifidobacterium breve*. Appl Environ Microbiol.

[78] Shkoporov AN, Efimov BA, Khokhlova EV, Steele JL, Kafarskaia LI, Smeianov VV (2008). Characterization of plasmids from human infant Bifidobacterium strains: sequence analysis and construction of *E. coli*-Bifidobacterium shuttle vectors. Plasmid.

[79] Lee JH, O’Sullivan DJ (2006). Sequence analysis of two cryptic plasmids from *Bifidobacterium longum *DJO10A and construction of a shuttle cloning vector. Appl Environ Microbiol.

[80] Fang S, Wang S, Liu Y, Zhu C, Wang S, Xu F (2026). Clinical safety and tolerability of *Bifidobacterium bifidum* BBi32: an 8-week randomized, double-blind, placebo-controlled trial with genomic and *in vitro* corroboration. Food Sci Nutr.

[81] Sgorbati B, Scardovi V, Leblanc DJ (1982). Plasmids in the genus Bifidobacterium. J Gen Microbiol.

[82] Fries M, Jung HI, Perham RN (2003). Reaction mechanism of the heterotetrameric (alpha2beta2) E1 component of 2-oxo acid dehydrogenase multienzyme complexes. Biochemistry.

[83] Newstead SL, Watson JN, Bennet AJ, Taylor G (2005). Galactose recognition by the carbohydrate-binding module of a bacterial sialidase. Acta Crystallogr D Biol Crystallogr.

[84] Francl AL, Hoeflinger JL, Miller MJ (2012). Identification of lactose phosphotransferase systems in *Lactobacillus gasseri* ATCC 33323 required for lactose utilization. Microbiology (Reading).

[85] Caminero A, Guzman M, Libertucci J, Lomax AE (2023). The emerging roles of bacterial proteases in intestinal diseases. Gut Microbes.

[86] Supuran CT (2023). Carbonic anhydrase versatility: from pH regulation to CO_2_ sensing and metabolism. Front Mol Biosci.

[87] Zhang S, Li P, Zhang X (2024). Lipoteichoic Acid from Heyndrickxia coagulans HOM5301 modulates the immune response of RAW 264.7 macrophages. Nutrients.

[88] Li X, Wang G, Zhang M (2026). Anti-inflammatory potential of teichoic acids from Lactiplantibacillus plantarum AR113 and their effects on host immune responses. Food Bioscience.

[89] Turroni F, Bottacini F, Foroni E (2010). Genome analysis of *Bifidobacterium bifidum* PRL2010 reveals metabolic pathways for host-derived glycan foraging. Proc Natl Acad Sci U S A.

[90] Saier MH, Reddy VS, Moreno-Hagelsieb G (2021). The Transporter Classification Database (TCDB): 2021 update. Nucleic Acids Res.

[91] Nayfach S, Shi ZJ, Seshadri R, Pollard KS, Kyrpides NC (2019). New insights from uncultivated genomes of the global human gut microbiome. Nature.

[92] Pasolli E, Asnicar F, Manara S (2019). Extensive unexplored human microbiome diversity revealed by over 150,000 genomes from metagenomes spanning age, geography, and lifestyle. Cell.

[93] Almeida A, Mitchell AL, Boland M (2019). A new genomic blueprint of the human gut microbiota. Nature.

[94] Tett A, Huang KD, Asnicar F (2019). The *Prevotella copri* complex comprises four distinct clades underrepresented in westernized populations. Cell Host Microbe.

[95] Karcher N, Pasolli E, Asnicar F (2020). Analysis of 1321 Eubacterium rectale genomes from metagenomes uncovers complex phylogeographic population structure and subspecies functional adaptations. Genome Biol.

[96] Shoer S, Reicher L, Zhao C, Pollard KS, Pilpel Y, Segal E (2024). Pangenomes of human gut microbiota uncover links between genetic diversity and stress response. Cell Host Microbe.

[97] Andreu-Sánchez S, Blanco-Míguez A, Wang D (2025). Global genetic diversity of human gut microbiome species is related to geographic location and host health. Cell.

[98] Vinjamuri A, Davis JCC, Totten SM (2022). Human milk oligosaccharide compositions illustrate global variations in early nutrition. J Nutr.

[99] Derrien M, Mikulic N, Uyoga MA (2023). Gut microbiome function and composition in infants from rural Kenya and association with human milk oligosaccharides. Gut Microbes.

[100] Stražar M, Temba GS, Vlamakis H (2021). Gut microbiome-mediated metabolism effects on immunity in rural and urban African populations. Nat Commun.

[101] de Goffau MC, Jallow AT, Sanyang C (2022). Gut microbiomes from Gambian infants reveal the development of a non-industrialized Prevotella-based trophic network. Nat Microbiol.

[102] Carvalho MJ, Sands K, Thomson K (2022). Antibiotic resistance genes in the gut microbiota of mothers and linked neonates with or without sepsis from low- and middle-income countries. Nat Microbiol.

[103] Mudhluli TE, Kujawska M, Mueller J (2024). Exploring the genomic traits of infant-associated microbiota members from a Zimbabwean cohort. BMC Genomics.

[104] Katoh T, Ojima MN, Sakanaka M, Ashida H, Gotoh A, Katayama T (2020). Enzymatic adaptation of *Bifidobacterium bifidum* to host glycans, viewed from glycoside hydrolyases and carbohydrate-binding modules. Microorganisms.

[105] Liu J, Li W, Yao C, Yu J, Zhang H (2022). Comparative genomic analysis revealed genetic divergence between *Bifidobacterium catenulatum* subspecies present in infant versus adult guts. BMC Microbiol.

[106] Wu Q, Li W, Kwok LY, Lv H, Sun J, Sun Z (2024). Regional variation and adaptive evolution in *Bifidobacterium pseudocatenulatum*: insights into genomic and functional diversity in human gut. Food Res Int.

[107] Wang J, Qian X, Li Q (2026). Bacteriocin gene-mediated ecological adaptation of *Bifidobacterium breve* in the adult human gut. Cell Genom.

[108] Ioannou A, Knol J, Belzer C (2021). Microbial glycoside hydrolases in the first year of life: an analysis review on their presence and importance in infant gut. Front Microbiol.

