# Supplementary material for: Genome-scale insights into the metabolic landscape and evolutionary development of Bifidobacterium bifidum
Source: Microbiome Res Rep. 2026 Aug 12;5(3):18. doi: 10.20517/mrr.2026.19 (PMC13678547; doi:10.20517/mrr.2026.19)
Supplement: Supplementary file 1 [file mrr-5-3-18-SupplementaryMaterials.zip › Supplementary Figures.pdf]

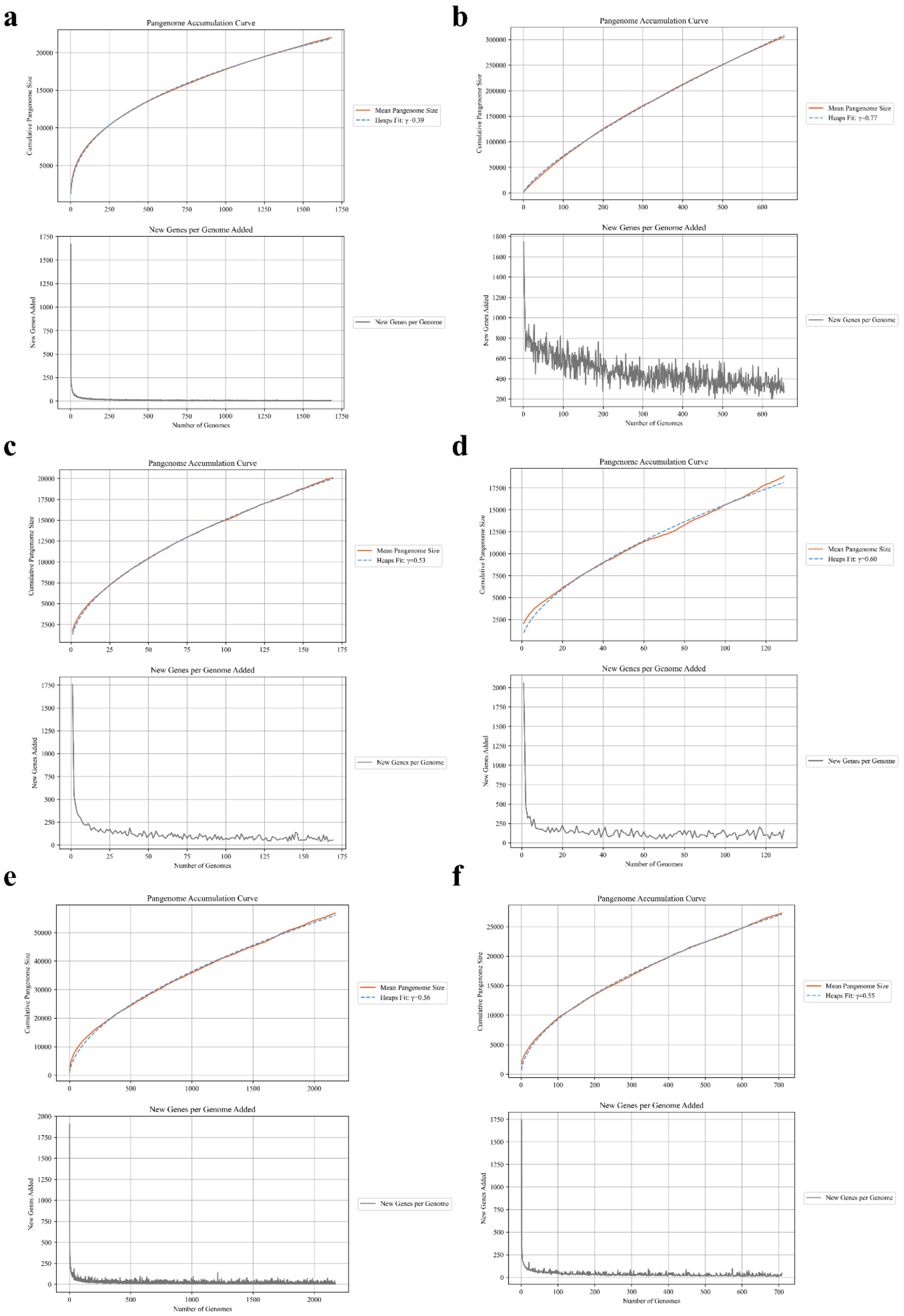

Supplementary Figure 1. Pangenome growth of HBS. Pangenome accumulation curves were generated from the comprehensive (a) *B. adolescentis* dataset [1] and high-quality

genomic sequences retrieved from NCBI for the other five HBS: *(b) B. breve*, *(c) B. catenulatum*, *(d) B. dentium*, *(e) B. longum*, and *(f) B. pseudocatenulatum*. For each species, the growth of its pangenome was correlated with increases in the number of genes (upper graphs) and the identification of new genes (lower graphs). In the graphs, the X-axis represents the number of genomes of the species, while the Y-axis is the variable to indicate the increase in the pangenome.

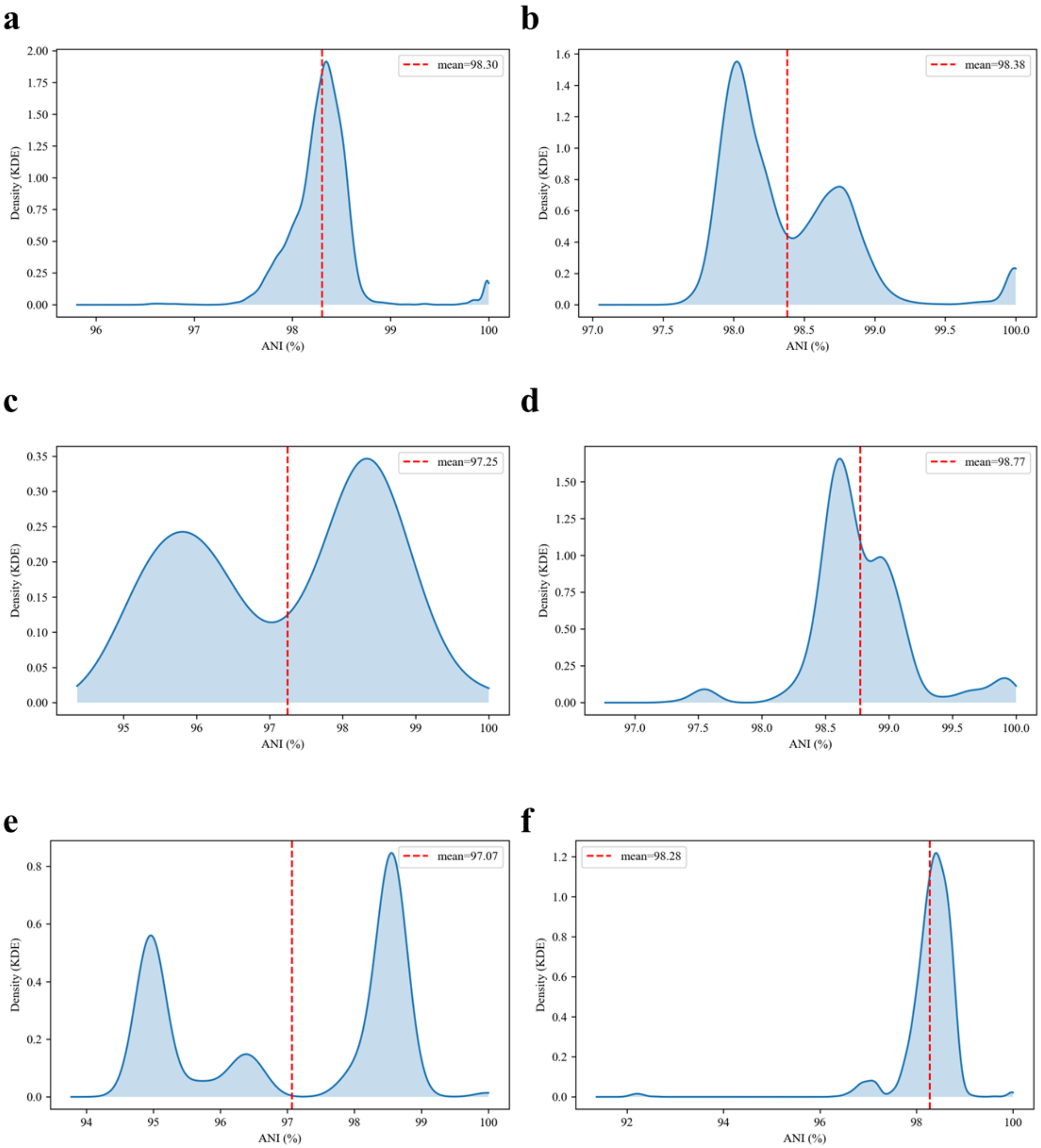

Supplementary Figure 2. Genetic distances within other HBS genomic clusters. Each panel (from a to f) represents a density plot of ANI values obtained from pairwise genome

comparisons across the other six HBS: *(a) B. adolescentis*, *(b) B. breve*, *(c) B. catenulatum*, *(d) B. dentium*, *(e) B. longum*, and *(f) B. pseudocatenulatum*. The mean ANI value and range are indicated for each cluster.

Supplementary Figure 3

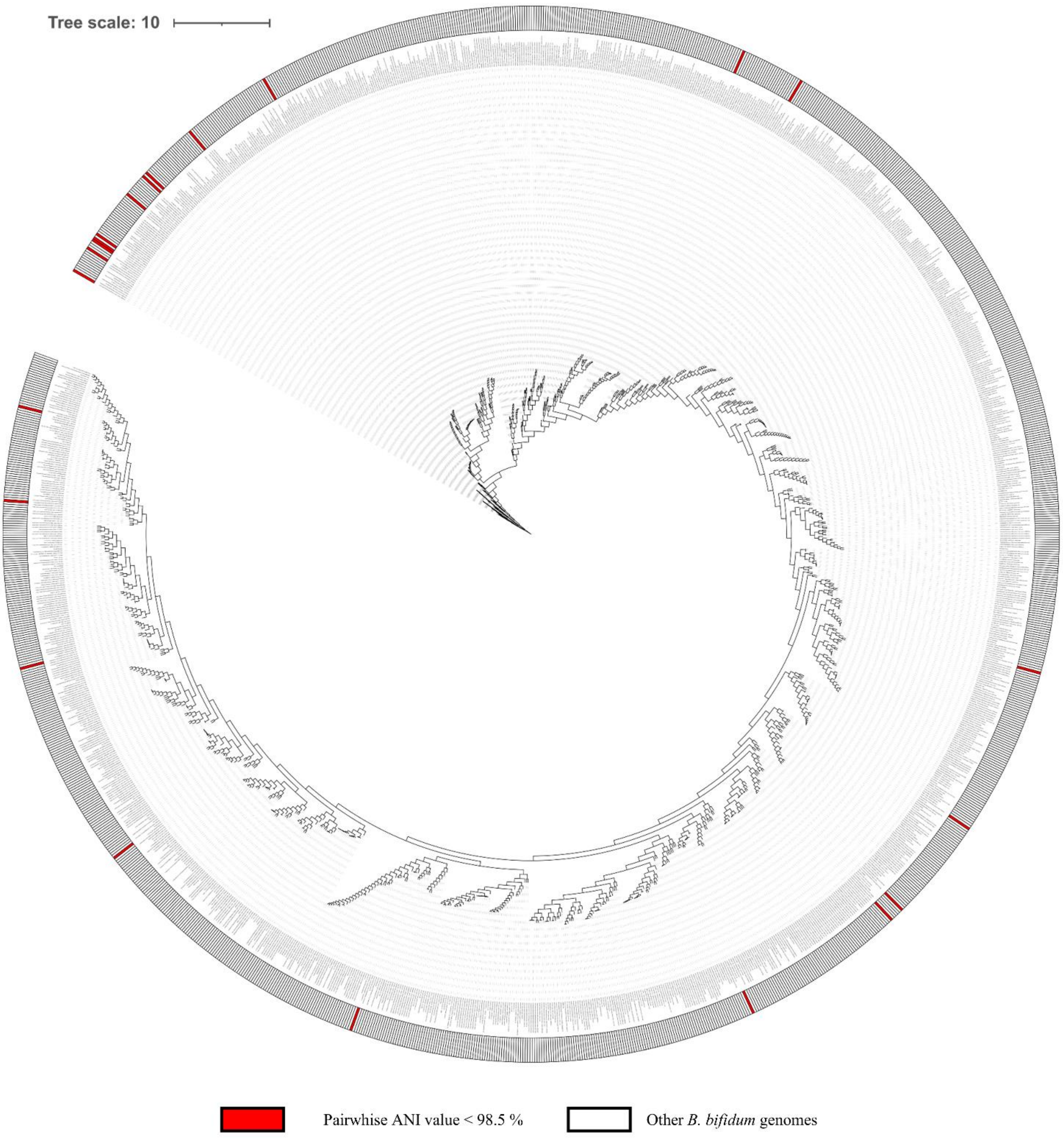

Supplementary Figure 3. ANI-based phylogenomic tree of the 1,351 B. bifidum genomes analysed in this study. The outer ring highlights in red the 22 genomes exhibiting ANI values

below 98.5%, illustrating their scattered distribution across the tree rather than a discrete phylogenetic clustering.

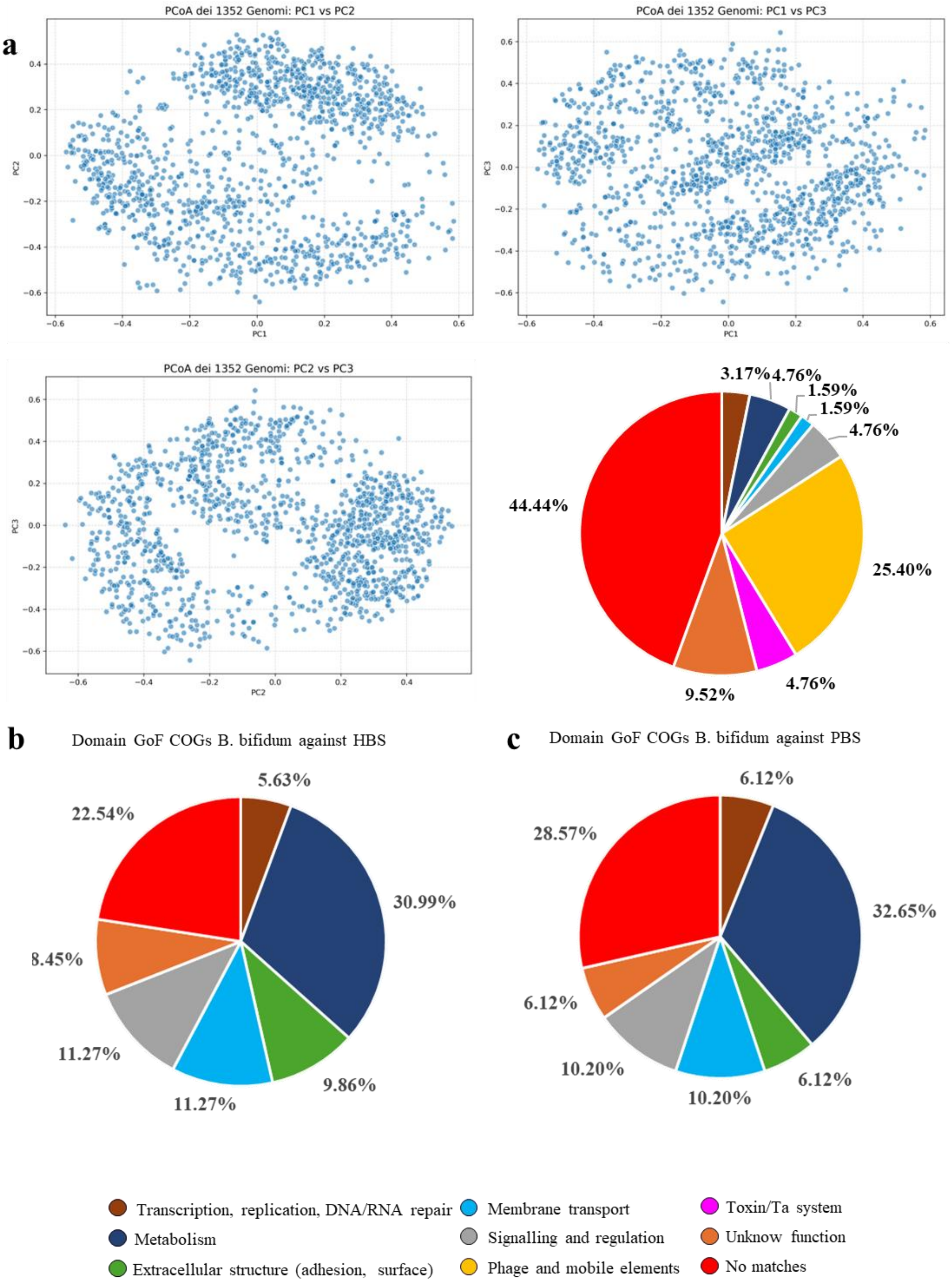

Supplementary Figure 4. Analysis of the evolutionary path of B. bifidum through GoF and/or LoF events. Panel (a) shows the distribution of the genomes of the B. bifidum dataset in the PCoA, depending on the presence of the 113 GoF/LoF COGs biparting the species and the subdivision of the COGs according to the type of protein domain identified in the corresponding amino acid sequence. Panels (b) and (c) show the subdivision of the COGs based on the protein domains of the COGs with significant GoF events towards the other HBS and towards the other PBS, respectively.

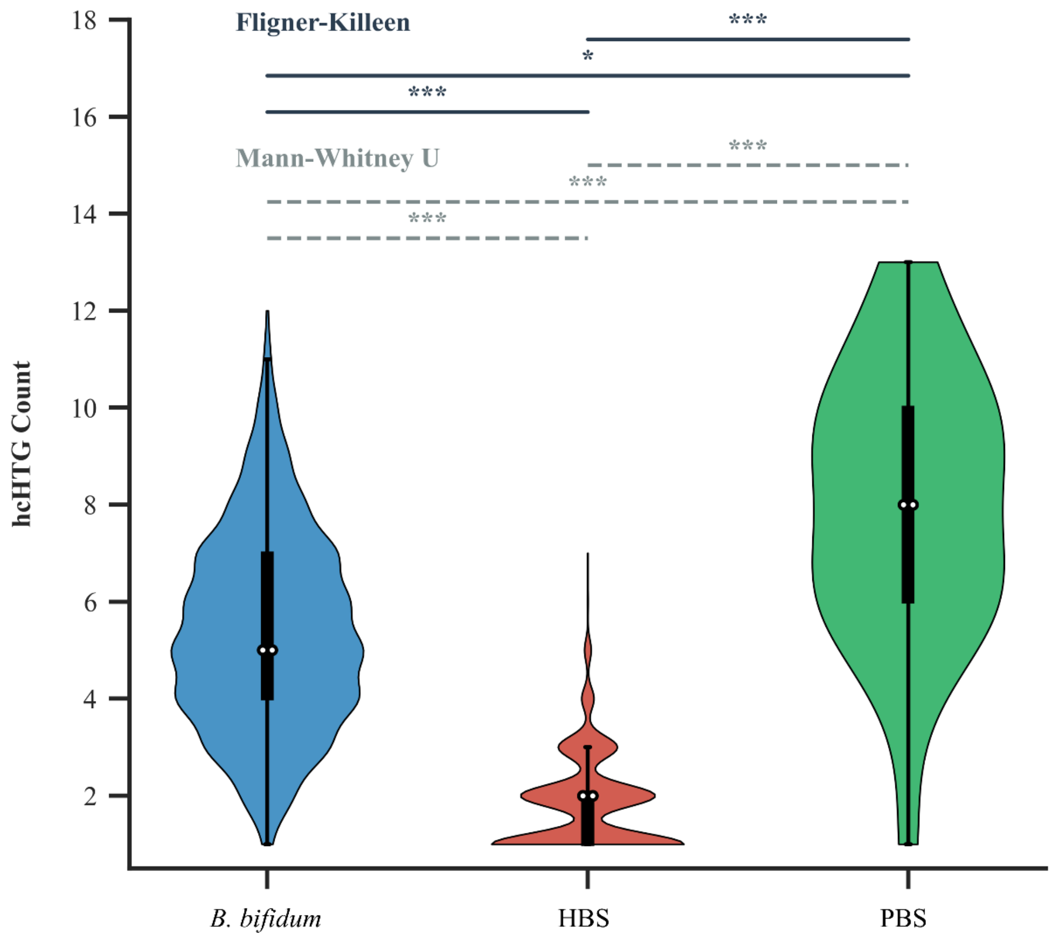

Supplementary Figure 5. hcHTG counts varied substantially across the three genomic groups considered, B. bifidum, HBS, and PBS. Pairwise differences in central tendency were tested with the Mann-Whitney U test, while differences in dispersion were assessed using the Fligner-Killeen test for homogeneity of variances. Both sets of p-values were corrected for multiple comparisons using the Benjamini-Hochberg FDR procedure. The asterisks above

each bracket indicate the significance level according to the code. (* $p<0.05$, ** $p<0.01$, *** $p<0.001$).

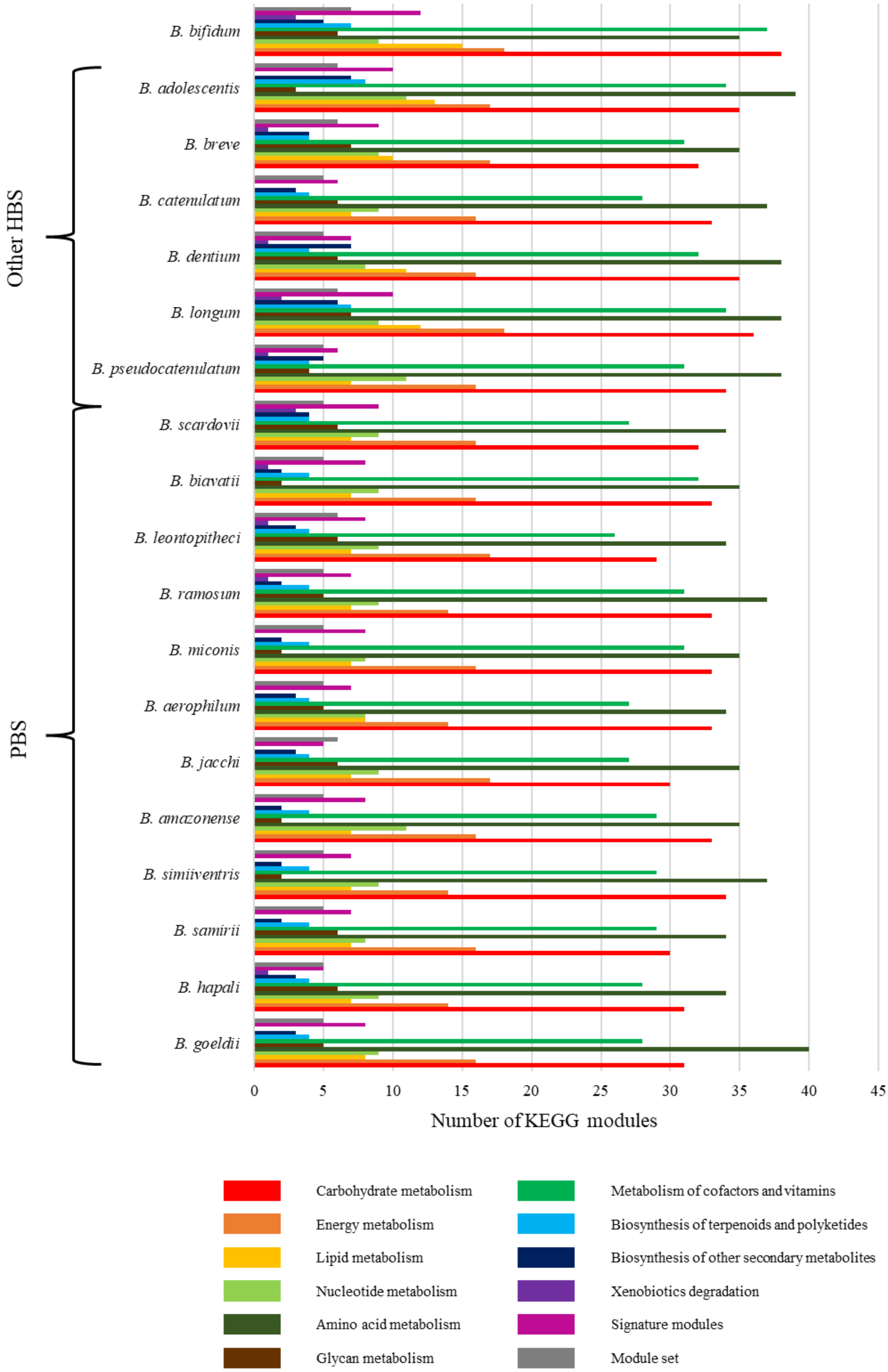

Other HBS
PBS
B. bifidum
B. adolescentis
B. breve
B. catenulatum
B. dentium
B. longum
B. pseudocatenulatum
B. scardovii
B. biavatii
B. leontopitheci
B. ramosum
B. miconis
B. aerophilum
B. jacchi
B. amazonense
B. simiiventris
B. samirii
B. hapali
B. goeldii
0
5
10
15
20
25
30
35
40
45
Number of KEGG modules
Carbohydrate metabolism
Energy metabolism
Lipid metabolism
Nucleotide metabolism
Amino acid metabolism
Glycan metabolism
Metabolism of cofactors and vitamins
Biosynthesis of terpenoids and polyketides
Biosynthesis of other secondary metabolites
Xenobiotics degradation
Signature modules
Module set

Supplementary Figure 6. KEGG metabolic modules identified in the different Bifidobacterium species present in the study. All KEGG metabolic modules with a non-zero completeness percentage were considered for comparison. The modules are rearranged and grouped according to their metabolic macroclass.

REFERENCES

1. Selleri E, Tarracchini C, Petraro S, Mancabelli L, Milani C, Turroni F, et al. Assessment of genome evolution in *Bifidobacterium adolescentis* indicates genetic adaptation to the human gut. *mSystems* 2026;11. [DOI: 10.1128/msystems.01173-25]
